# Patient-Specific Lattice Implants for Segmental Femoral and Tibial Reconstruction (Part 1): Defect Patterns, Fixation Strategies and Reconstruction Options—A Review

**DOI:** 10.3390/biomimetics11020128

**Published:** 2026-02-10

**Authors:** Mansoureh Rezapourian, Anooshe Sadat Mirhakimi, Mahan Nematollahi, Tatevik Minasyan, Irina Hussainova

**Affiliations:** 1 Department of Mechanical and Industrial Engineering, Tallinn University of Technology, 19086 Tallinn, Estonia; tatevik.minasyan@taltech.ee (T.M.); irina.hussainova@taltech.ee (I.H.); 2 Department of Mechanical Engineering, McMaster University, Hamilton, ON L8S 4L7, Canada; mirhakia@mcmaster.ca; 3 Department of Technical Physics, University of Eastern Finland, 70210 Kuopio, Finland; mahan.nematollahi@uef.fi

**Keywords:** patient-specific implants, segmental long bone defects, CT-based surgical planning, porous bone scaffolds, fixation strategies, mechanobiology, segmental femoral and tibial defects, critical-sized bone defects, 3D-printed porous scaffolds, lattice implants, additive manufacturing in orthopaedics, scaffold-guided bone regeneration, locking plate fixation, stress shielding, femoral and tibial reconstruction, load-bearing, personalized bone tissue engineering

## Abstract

This first part of a two-part review examines how Computed Tomography(CT)-based, additively manufactured (AM) porous implants are used to reconstruct large segmental defects of the femur and tibia. We focus on lightweight patient-specific lattice implants, architected cages, and modular porous constructs that incorporate engineered porosity into the load-bearing structure and are deployed with plate-, nail-, or external-fixator-based stabilization. We show how defects are described and classified by size, morphology, and anatomical subsegment; how these descriptors influence fixation choice and the resulting mechanical environment; and where along the femur and tibia porous implants have been applied in clinical and preclinical settings. Across the literature, outcomes appear to depend most strongly on defect morphology and local biology, while fixation feasibility and construct behavior vary by subregional anatomy. Most reported constructs use Ti6Al4V porous architectures intended to share load with fixation, reduce stress shielding, and provide a regenerative space for graft and tissue ingrowth. Finite element analyses (FEA) and bench-top studies consistently indicate that lattice architecture, relative density (RD), and fixation concept jointly control stiffness, micromotion, and fatigue-sensitive regions, whereas early animal and human reports describe promising incorporation and functional recovery in selected cases. However, defect descriptors, fixation reporting, boundary conditions, and outcome metrics remain diverse, and explicit quantitative validation of simulations against mechanical or in vivo measurements is uncommon. Most published work relies on simulation and bench testing, with limited reporting of biological endpoints, leaving a validation gap that prevents direct translation. We emphasize the need for standardized defect and fixation descriptors, harmonized mechanical and modeling protocols, and defect-centered datasets that integrate anatomy, mechanics, and longitudinal outcomes. Across the 27 included studies (may be counted in more than one group), simulation and mechanical testing are reported in 19/27 (70%) and 15/27 (56%), respectively, while in vivo studies (preclinical or clinical) account for 9/27 (33%), highlighting a validation gap that limits translation. Part 2 (under review); of these two series review paper; Patient-Specific Lattice Implants for Segmental Femoral and Tibial Reconstruction (Part 2): CT-Based Personalization, Design Workflows, and Validation-A Review; extends this work by detailing CT-to-implant workflows, lattice design strategies, and methodological validation.

## 1. Introduction

Segmental diaphyseal defects in the femur and tibia are among the most challenging issues in limb reconstruction. Even with modern techniques such as distraction osteogenesis and Masquelet’s induced membrane method, these defects remain difficult to address. When using vascularized bone grafts and megaprostheses, it is essential to understand that achieving union often requires an extended treatment period. This time investment is essential for optimal recovery. Recent reviews of post-traumatic tibial defects and induced membrane reconstructions report complication rates that include infections, malalignment, and the need for secondary procedures, despite high overall union rates, highlighting the biological and mechanical fragility of these reconstructions [1,2,3]. This ongoing challenge has generated increasing interest in methods that can effectively combine immediate mechanical stability with a bone-friendly environment in large femoral and tibial gaps.

Additive Manufacturing (AM) has transitioned from experimental use to routine application for Patient-Specific Implants (PSIs) in oncologic surgery and complex trauma. Case series and systematic reviews on custom 3D-printed devices for diaphyseal and distal tibial defects indicate that PSIs can effectively restore limb length and alignment. They also simplify the reconstruction of irregular defects and reduce the need for large allografts. However, it is important to note that the follow-up periods in these studies remain relatively short, and complication profiles vary widely [4,5].

Recently, personalized lattice or truss-like implants with integrated screw holes and stems have emerged as alternatives to conventional cages and megaprostheses, showing early osseointegration and improved functionality in selected large defects [6]. These advancements clearly showcase the effectiveness of CT-planned, AM-fabricated constructs in integrating fixation, defect filling, and joint-preserving reconstruction into a single device.

Simultaneously, architected porous materials have evolved into a rich design space for bone substitutes. Recent studies show that bone-mimic lattices can be tailored to match bone in stiffness, strength, fatigue life, and permeability by adjusting unit cell type, Relative Density (RD), and architectural gradients, rather than altering the bulk alloy [7,8,9,10]. Such lattices provide interconnected porosity for vascular and bone ingrowth while enabling controlled stress transfer to the host bone. This approach directly addresses longstanding issues of stress shielding and aseptic loosening in dense intercalary grafts and megaprostheses [11].

Advancements in clinical practices and materials rely heavily on image-based planning and numerical modeling. Patient-specific finite element (FE) workflows now enable the integration of CT-based femoral and tibial geometries with specific loading conditions to evaluate implant stiffness, screw trajectories, and bone strain distributions before surgery [12,13]. Recent studies have extended this concept to AM lattice implants, employing FE analysis and topology optimization to develop design guidelines for distal femoral reconstructions or to validate patient-specific lattice segments used in femoral defects against experimental data [14,15]. However, the existing data remain incomplete and diverse. Different groups focus on defect classification, defect fixation strategies, lattice architecture, or modeling methodologies, and only a few integrate all these elements into a unified defect-to-design workflow [16].

This first part of our two-part review focuses on segmental defects of the femur and tibia that are reconstructed with CT-based, AM-fabricated porous implants. We concentrate on reconstructions that incorporate engineered porosity into the load-bearing structure. This includes patient-specific lattice implants, modular porous blocks, and architected cages, which are used in conjunction with plates, nails, or external fixators. Common bone graft substitutes and solid, non-porous megaprostheses are not the focus of this two-part review study. Part I aims to achieve three objectives: to outline how clinical series and preclinical models characterize defect archetypes and how these characterizations affect fixation strategies and the mechanical environment; to create an overview of anatomical and fixation information regarding documented femoral and tibial reconstructions, with a focus on the use of porous implants in different segments and sizes of defects; and to combine the mechanical proof from both simulations and experiments that backs up these concepts. This framework aims to provide a mechanobiology context for designing and interpreting PSLIs used in segmental reconstruction of the femur and tibia, while the companion Part 2 review addresses CT-to-implant workflows, lattice design strategies, additive manufacturing routes, and the detailed methodological reporting of experimental, computational, biological, and clinical validation.

This review follows a structured narrative approach. We performed targeted searches in PubMed/MEDLINE and Google Scholar (and Scopus/Web of Science where available) from [2013–2025] using combinations of keywords, including patient-specific, lattice, porous, TPMS, AM/3D printing, segmental defect, femur, tibia, reconstruction, and fixation methods. Studies were included if they described patient-specific lattice/porous implants for femoral or tibial segmental defect reconstruction and provided sufficient details to classify the defect archetype and the fixation/interface concept. Studies were excluded if they focused only on the lattice without an implant reconstruction context or lacked key reporting required for classification. Where multiple publications reported overlapping cases or the same implant series, we avoided double counting in total summaries and extracted quantitative fields from the most complete report.

## 2. Segmental Defect Typing and Implant–Defect Interactions

**Figure 1:**
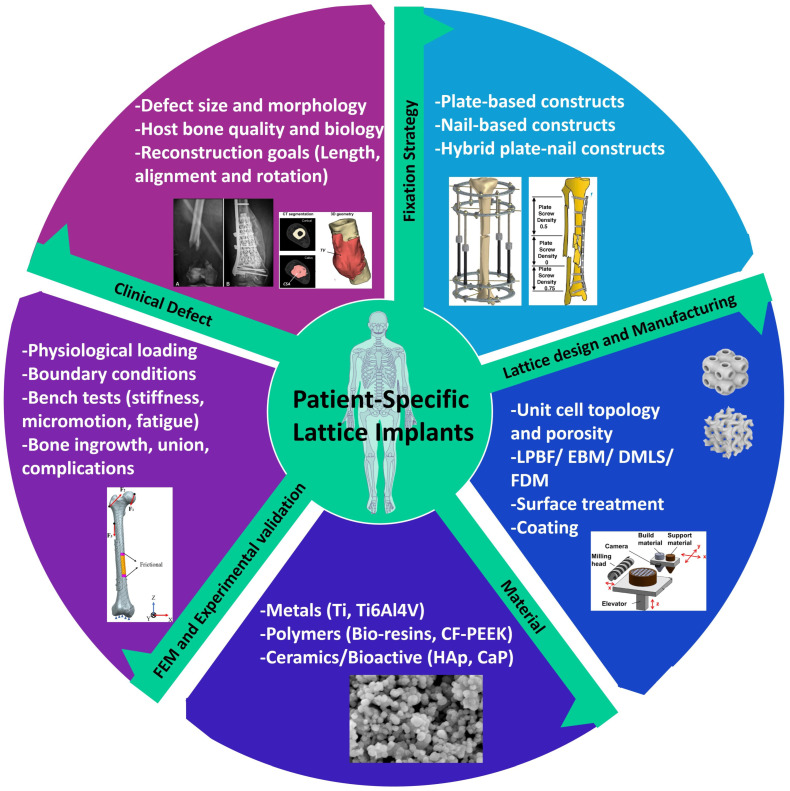
Multi-domain framework for PSLIs in long bone reconstruction. Characterization of clinical defects using CT and X-ray images, along with defect illustrations [17,18]. Fixation strategy examples showing plate-based, nail-based, and hybrid constructs [19,20]. Material microstructures illustrating metallic, polymeric, and bioactive ceramic systems [21,22]. Lattice design and manufacturing workflow for architected scaffolds, including unit cell topologies and AM process steps [23,24,25]. Finite element Method (FEM) and experimental validation schematics showing physiological loading and bench tests [26,27]. The central circle highlights that PSLIs lie at the intersection of these five domains, and achieving robust outcomes requires coordinated decisions across all domains.

Reconstruction of segmental defects in the femur and tibia involves several interconnected design domains, including defect morphology, fixation strategy, material selection, lattice architecture, and validation methods. Figure 1 illustrates this diverse framework and emphasizes how PSLIs are influenced by the clinical defect and implant concepts, which will be further discussed in the subsections on defect typing and implant effects.

### Defect Typing in Femur and Tibia: Rule of Geometry and Size

Segmental defects in the femur and tibia can differ in healing outcomes. Across the reviewed studies, outcomes appear to depend most strongly on defect morphology and local biological conditions (e.g., circumferential bone loss, soft tissue compromise, and infection). In contrast, anatomical factors such as the involved bone and subregional location can still influence fixation feasibility and healing [28]. Across studies, a wide range of reconstructive techniques has been employed, including Ilizarov bone transport, the Masquelet induced membrane procedure, vascularized iliac crest or fibular grafts, irradiated autografts, and diamond concept reconstructions [29]. Union rates differed significantly across these methods [30]. In large and complex defects, approximately 50% of cases achieved union [28], whereas more minor or more biologically favorable defects often demonstrated complete union [28]. Healing times varied significantly, ranging from approximately 4 months in select cases with strong graft biology to over 18 months in extensive defects treated with staged distraction osteogenesis [30]. Defects that are larger, especially those measuring between 10 and 18 cm, have been consistently associated with less favorable outcomes [31,32]. This includes extended consolidation periods and increased rates of delayed union or nonunion [31]. These cases often needed additional interventions. In addition to morphology and host factors, subregional anatomy also influenced outcomes [33,34,35,36]. Defect-related factors were crucial in predicting healing outcomes. Large defects with compromised soft-tissue envelopes, infections, or previous failed reconstructions exhibited significantly poorer healing potential and higher complication rates [37,38,39]. These complex defects often required staged or combined reconstruction strategies in order to attain optimal biological and mechanical stability [40,41,42]. However, the need for reoperation remained high, particularly in significant or infected defects where biological and structural challenges were most pronounced [43]. This perspective highlights that the primary factors, including defect size, location, host biology, and the status of any existing infections, influence the healing process deeply [44,45].

**Figure 2:**
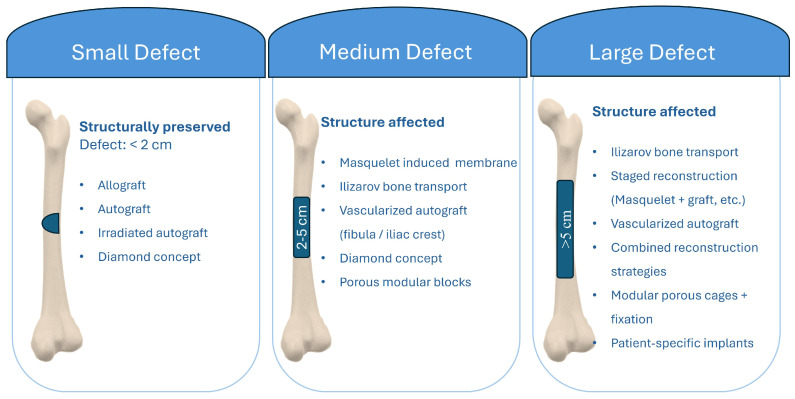
Size-based classification of segmental long bone defects and corresponding reconstructive options; small defects < 2 cm, intermediate defects 2–5 cm, large defects > 5 cm.

Figure 2 summarizes size-based classification of segmental femoral defects into small (<2 cm), intermediate (2–5 cm), and large (>5 cm) categories. For each class, the figure outlines commonly used reconstructive solutions. These range from shortening, cement augmentation, and grafting for small defects to multi-modal combinations of vascularized autografts, bone transport, adjunctive cement, and patient-specific implants for very large defects. Defect size was grouped as a practical way to structure the reviewed literature, not as a clinical guideline, because treatment pathways and outcome risks shift clearly when defect length exceeds a few centimeters and biological or soft tissue compromise becomes more frequent.

To complement the conceptual size-based grouping in Figure 2, we additionally summarized the included studies by plotting defect size class versus anatomical subsegment for selected studies in Figure S1 and including a characterization flowchart of reconstruction pathways for segmental long bones in Figure S2 (Supplementary Materials). This stratified distribution highlights an anatomical sampling bias in the current literature, with most included studies focusing on femoral defects (particularly diaphyseal cases) and a predominance of large defects (>5 cm), whereas tibial segmental defect studies are rarely represented in the dataset.

## 3. Fixation Strategy, Mechanical Environment, and Implant Longevity

Fixation strategy is a principal determinant of healing outcomes in long bone defects, including segmental and metaphyseal defects of the femur and tibia [46,47,48,49]. Growing clinical and biomechanical case studies show that both the choice of fixation construct and its detailed mechanical behavior critically shape bone regeneration [50]. Traditional fixation methods such as locked plating, intramedullary (IM) nailing, and circular external fixation are being significantly improved through the use of hybrid configurations (such as nail–plate constructs), biologically based techniques (including the Masquelet method and bone transport), and emerging patient-specific or implantable scaffold constructs [51,52].

To quantitatively and consistently relate mechanobiology to design choices, the PSLI literature can be analyzed through five recurring mechanical characteristics: defect-/gap-level motion (in mm), implant–bone interface micromotion (in μm), local strain conditions within bone/scaffold (as a percentage or microstrain), construct stiffness along with load sharing between fixation and scaffold (in N/mm or kN/mm), and imaging-based proxies for interface stability (such as BIC/BII).

Within the reviewed studies, Liu et al. [24] reported that a relative displacement exceeding 150 μm encourages the formation of fibrous tissue while inhibiting bone growth, and they further discussed scaffold-level bone strain of approximately 1–5% (with 1% noted for early-stage bone formation) as facilitative to bone formation in porous titanium scaffolds. Using a complementary mechanostat-style framing in patient-specific implant analysis, Wong et al. [53] referenced a damage strain level of approximately 4000 μϵ, above which microdamage may delay union or contribute to nonunion, and interpreted interface strains predominantly below this level as favorable in the immediate postoperative stage while noting limitations in representing the micro-porous interface and subsequent tissue evolution in macro-scale FE models [53]. Karuppudaiyan et al. [54] reported scaffold displacement magnitudes of 0.0031–0.012 mm across porosity variants (intact femur: 0.002 mm) and explicitly linked these differences to relative motion and stress shielding considerations [54]. Entezari et al. [55] described how rigid plating mainly transfers load through the plate, which can limit load on the scaffold and risk stress shielding. High strain energy indicates excessive movement, exceeding normal limits. This highlights the balance between the need for stable interfaces early on to reduce micromotion and the need for load transfer later to prevent ongoing stress shielding. This explanation aligns with the arguments of stress shielding, framed as a reduction in strain and a mismatch in stiffness distribution, which are used to support strategies involving porous and/or graded stiffness [24,56]. Multiple studies have recognized the dependence on time: the process of bone ingrowth and remodeling contributes to a gradual increase in interface stability, leading to a reduction in interface micromotion that ultimately ceases as stability improves [26,57]. Zhang et al. [57] also presented design parameters, including a pore size of 400–600 μm, a strut diameter of 240–320 μm, and a porosity of 60–80%. They reported FE peak stresses at the interface between the implant and bone complex of 12.09 MPa under 1000 N, 3.09 MPa under 2000 N, and 37.21 MPa under 3000 N, while addressing micromotion in a mechanistic way without detailing its magnitude. Robust quantitative mechanobiology connections are established in studies that explicitly address stiffness and strain.

Blázquez-Carmona et al. [18] examined the evolution of load sharing over time and changes in stiffness, finding that the external fixator had a stiffness of Kf = 593 N/mm, carried more than 90% of the force during the first two weeks, and experienced a reversal in load sharing after approximately 30 days, with callus stiffness rising from around 650 N/mm at day 40 to about 7 kN/mm by day 60. They also identified a remodeling transition occurring near 200 days. Pobloth et al. [58] directly compared different fixation stiffness regimes, including a load-sharing plate with a stiffness of approximately 555 N/mm and a stress shielding plate with a stiffness of about 2857 N/mm, alongside scaffold stiffness ranging from 0.20 to 3.09 kN/mm, and pore-level principal strain magnitudes of around 0.23–0.6% in autograft-filled pores and approximately 0.65–1.3% in callus-filled pores. They also elaborated on bone formation within porous implants, noting a strain range of 5% to 0.04%, and discussed resorption levels below 0.04% under finite element loads of 1372 N in compression and 86 N in bending. Chang et al. [27] similarly used a strain-related design criterion by treating 4000 μϵ as an upper bound for favorable bone growth around the interface, reporting measured strains of 2046–2253 μϵ as conducive to bone growth and providing simulation and experiment comparisons (e.g., 1752.6 vs. 1687.9 μϵ, 2046.4 vs. 2133.7 μϵ, 2252.57 vs. 2292.55 μϵ), while linking geometry/material choices to stress shielding and osseointegration outcomes (e.g., pillar diameter 0.9 mm associated with implant modulus 14.99–15.88 GPa and strains exceeding 4000 μϵ; bone cement modulus 2.65 GPa; intraluminal growth > 79.8%).

However, the quality of the interface can be effectively quantified through imaging indicators even without direct measurements of micromotion magnitudes. Obaton et al. [59] used a 10 μm reference limit (with XCT pixel size 6.8 μm), reported bone–interface implant (BII = 3.3 μm) and bone–implant contact (BIC = 95.4% (with 50–80%) and discussed stiffness mismatch (cortical bone 25–30 GPa vs alloy 38 GPa) as a contributor to stress concentration/stress shielding considerations.

Several studies provide quantitative inputs that mainly describe the mechanical context without offering a direct mechanobiological interpretation. This involves structured weight-bearing protocols, such as partial weight bearing set at 35 kg for a duration of 6 weeks, followed by an advancement to full weight bearing in the succeeding 6 weeks [17]. The reported scaffold design features envelopes with porosities reaching up to 82.7%, compressive strengths ranging from 1.76 to 9.34 MPa, and structural moduli between 52.2 and 212 MPa [60]. Stiffness gradient architectures have been described with pore sizes of 500 μm, porosities of 84.22/77.68/68.45%, and corresponding moduli of 0.55/1.40/3.49 GPa [61]. Other reports emphasize stress shielding through geometry and loading definitions without explicit micromotion analysis, such as ATS dimensions of 8 × 8 × 5.8 mm, strut sizes of 200–500 μm, porosities of 50% versus 62%, cyclic loading at 150 N, and finite element pressure of 1.25 MPa [62]. In addition, failure metrics from printed femur models have been reported, with fracture signs at approximately 1.9% strain and failure at 208.58 MPa and 1.99% strain [63].

FE analysis and experimental studies consistently demonstrate that optimal fixation achieves a balance between sufficient axial and torsional stability and controlled micromotion that stimulates callus formation [15,52,64,65]. Structures that facilitate controlled load sharing via compliant scaffold architectures, optimized plate configurations, or combined nail–plate systems create a more physiologic mechanical environment and have been associated with higher union rates and fewer reoperations in clinical cases [51,64,66,67]. Inadequate matching of construct stiffness to the local mechanical environment can lead to stress shielding, progressive bone loss, and late aseptic loosening, whereas overly flexible constructs might lead to fatigue failure or an inability to heal properly [68,69,70,71]. These results highlight that the design of fixation (fixation strategy) is not only a matter of structure but is essentially biological, as the stiffness of the construct and micromotion are tightly coupled to tissue differentiation, the maturation of callus, and implant longevity [58,72,73,74]. In the immediate period following surgery, femoral and tibial reconstructions successfully regain function compared to the state before the operation. Nonetheless, noticeable deficits in relation to the other limb continue to exist. Moreover, early complication rates are reported at around 3–7%, with failures often resulting in implant fractures, lag-screw cut-out, periprosthetic fracture, aseptic loosening, and nonunion [75,76,77]. Micromotion at the implant–bone interface is crucial for bone ingrowth. Excessive micromotion can lead to fibrous tissue formation and loosening, while insufficient micromotion may hinder osseointegration, resulting in long-term instability [78,79,80,81,82]. In long follow-up periods, stiffness mismatch and stress shielding significantly contribute to bone loss, aseptic loosening, and periprosthetic fractures. In contrast, utilizing low-modulus or porous/graded constructs effectively restores more physiological strain distributions, addressing these critical issues [68,69,70,71]. Some studies indicate that late infection rates range from approximately 2% to 14%, and mechanical failure rates vary between 2.5% and 30%, Additionally, cumulative revision rates are approaching 10% to 12% after 15 to 17 years [72,73,74,83].

Table 1 summarizes the quantitative mechanobiology metrics explicitly reported in the selected studies, linking mechanical measures (e.g., strain, micromotion, stiffness, and load sharing) to their biological interpretations and associated design levers. The complete details of the reported metrics are provided in Table S1 (Supplementary Materials).

These findings emphasize the interdependence of fixation strategy, mechanical environment, and implant longevity as critical design objectives. Moreover, they highlight the necessity for standardized reporting of Range of Motion (ROM), Patient-Reported Outcomes (PROs), complications, and quantitative mechanical metrics. Such standardization is essential for refining construct selection and enhancing long-term survivorship [72,73].

## 4. Anatomical and Fixation Context of Reported Implants

Segmental long bone defects arise in different anatomical locations with varying defect morphologies and host-bone conditions. These differences strongly influence the design, fixation, and evaluation of porous implants. A long, circumferential meta-diaphyseal defect in the distal femur presents very different mechanical and biological demands compared to a shorter diaphyseal gap in the tibia or a standardized defect in an ovine metatarsal, even if nominal defect length is similar. To keep this review clinically anchored, we therefore first harmonize how cohorts, imaging sources, defect archetypes, and fixation strategies are described across the literature, and use this [17,18,21,22,23,24,26,27,53,54,55,56,57,58,59,60,61,62,63,84,85,86,87,88,89,90] as a familiar backdrop for the subsequent sections on lattice architecture, fabrication, and simulation in the present Part I, and in detail in the companion Part 2 paper of this series.

Table 2 summarizes the essential details, while the complete study by study table is provided in Supplementary Table S1. The details—who, what, where, and how—provided for each study include: the reconstruction target (human patients, small or large animal models, or composite/cadaveric surrogates), what type of bone segment and defect archetype is considered (diaphyseal, metaphyseal, meta-diaphyseal; contained versus segmental or circumferential defects; gap length and morphology), and where/how the anatomy and void geometry are obtained (clinical CT, micro-CT, reference datasets, or synthetic models). We also record the selected stabilization concept and the translational maturity level (purely computational, bench-top, in vivo, or clinical).

Within this framework, patient-specific reconstructions in human femora and tibiae form one major subgroup. Lu et al. [90] and Benady et al. [91] design individualized porous Ti6Al4V implants directly on CT-derived long bone models and integrate them into patient-specific surgical workflows, while Zhang et al. [57] co-design cutting guides, stems, and porous segments for complex meta-diaphyseal reconstructions. A second subgroup comprises preclinical diaphyseal defect models in small and large animals, in which porous cylinders or meshes are implanted into standardized gaps under controlled loading to assess scaffold function and bone regeneration [57,87,88]. A third subgroup includes methodological and composite-bone studies in which synthetic femurs or biofidelic surrogates are used to calibrate FE models, test plate–scaffold constructs, or explore boundary conditions in a reproducible manner [21,84,92]. Materials-related work on surface treatments and coatings [56,93], together with broader reviews of patient-specific FEM and modeling practice [94,95], provides additional context for corrosion, osseointegration, and workflow standardization that underpins these implant designs. Across these classes, the imaging and modeling workflows follow a considerably similar pipeline. Most clinical and preclinical studies begin with CT or micro-CT Digital Imaging and Communications in Medicine (DICOM) data of the affected bone, sometimes complemented by Magnetic Resonance Imaging (MRI) in oncologic or infectious cases to refine resection margins and soft-tissue relationships [57,90,91].

Fixation strategy and boundary conditions are recorded in the overview because they define the mechanical environment in which the lattice operates and therefore modulate the interpretation of stress–strain patterns, micromotion, and failure risk. Several human and composite-femur studies analyze open-porous Ti scaffolds combined with lateral locking plates and screws, quantifying construct stiffness, stress distributions, and joint-surface displacement under stance-phase loading [84,90]. Benady et al. [91] and Zhang et al. [57] combine individualized porous segments with intramedullary nails, integrated stems, and/or plates, explicitly co-designing implant, guides, and fixation to restore alignment, length, and joint congruity in large segmental defects. In small- and large-animal models, fixation is often simplified—external fixators, lightweight plates, or in some cases no additional fixation—to isolate the contribution of scaffold stiffness, pore architecture, and material composition to bone regeneration [87,88]. At the other end of the spectrum, Shams et al. [21] and Baville et al. [92] use deliberately idealized “no-fixation” or abstract boundary conditions to prototype lattice concepts and compare inertia-relief versus spring or isostatic constraint formulations before applying them to more realistic constructs. Across these examples, assumptions such as bonded implant–bone interfaces, stance-phase or gait-based loading, and experimental calibration against composite specimens are made explicit [84,90,91,92].

**Figure 3:**
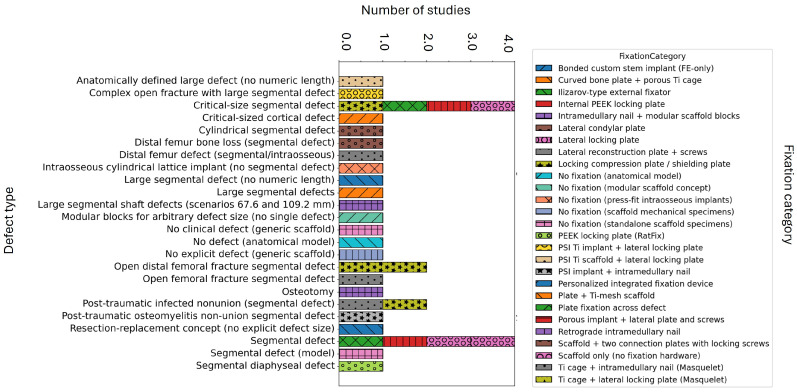
Representation of fixation strategies to segmental long bone defect types. Horizontal bars show the number of studies reporting each defect type, with colored and hatched segments indicating the corresponding fixation categories used.

In Table 2, we summarize, for each study, the bone segment and defect type, cohort level, imaging and segmentation approach, fixation strategy or implant-to-bone interface concept, and overall translational readiness. This overview shows that femoral diaphysis and tibial shaft models dominate the literature, while imaging and fixation choices vary widely, which makes direct comparison across studies difficult. The distribution of fixation constructs across the different defect archetypes is presented in Figure 3. In this review paper, bench refers to bench-top laboratory experiments performed outside of animals or patients (e.g., mechanical testing, phantom/Sawbones studies, or in vitro assays). The term in silico denotes computational-based studies, FE analyses, or numerical design workflows.

To enhance the qualitative representation presented in Figure 3 with quantitative specifics, we also present the frequency of fixation strategies based on defect size–anatomy combinations in Figure S3 (Supplementary Materials), which results from the studies outlined in Table 1. In general, plate-based constructs dominate most reported combinations, whereas intramedullary nails and hybrid plate–nail strategies are mainly observed in femoral diaphyseal reconstructions, particularly for larger defects. For this heatmap summary, we excluded entries that did not focus on segmental long bone defect reconstructions (e.g., no-defect models, generic scaffold studies, intraosseous implants) and those that lacked a clearly defined fixation construct.

Taken together, this overview highlights how the anatomical context and fixation selection influence the defined design space and the ability to compare results across studies. Reviewing Table 2 and Figure 3 has practical implications for how porous implants should be interpreted and designed. First, implant performance should not be discussed independently of anatomical site, defect types, and fixation strategy, because these elements jointly define the mechanical environment and determine how reported stresses, strains, and micromotion measures should be interpreted. Second, the variation in imaging pipelines, defect definitions, and fixation constructs imply that comparisons across studies are most meaningful when results are interpreted within clearly described subgroups, rather than as one combined literature. For future work, this supports a more explicit joint design logic in which fixation choice and implant architecture are selected together to match the intended clinical objective, including early stability, controlled load sharing, and long-term construct longevity. Finally, the overview highlights the need for consistent reporting of defect geometry, fixation construct, and boundary condition assumptions, as these variables determine whether findings can be translated into practical design rules.

## 5. Lattice Modeling and Architecture

Modern porous orthopedics emerged in the late 1960s–early 1970s, when it was recognized that bone could ingrow into stable porous metallic surfaces, enabling cementless long-term fixation. Early technologies—such as plasma-sprayed porous titanium, sintered fiber–metal meshes, and sintered cobalt–chromium bead coatings—defined the basic design principles for porous CoCr and Ti surfaces on hip and knee implants [96,97,98]. Subsequent reviews clearly established this rationale that these coatings reduce stress shielding, enhance surface roughness and friction, and promote robust osseointegration [97,99]. By the early–mid-1980s, porous-coated femoral stems and acetabular shells for cementless Total Hip Arthroplasties (THAs), together with porous-coated anatomic knee systems, were in routine use, with Engh, Bobyn, and others documenting bone ingrowth and the importance of press-fit mechanics [100,101]. In the late 1990s–2000s, these ideas developed into three-dimensional porous formations like highly porous tantalum metaphyseal cones (trabecular metal), which combined high porosity, relatively low stiffness, and high friction to stabilize large cavitary and segmental defects in femoral and tibial metaphyseal bone during complex revision Total Knee Arthroplasty (TKA) [102,103].

### From Coatings to Load-Sharing Lattice Architectures for Long Bone Reconstruction

Modern long bone reconstruction has evolved from surface porosity to advanced load-bearing lattices with adjustable permeability. Lattice scaffolds intended for the repair of long bone defects, especially in the femur and tibia, are structured porous materials developed with either periodic or random geometries [21,104,105,106,107,108]. These structures are engineered to provide mechanical support at both cortical and trabecular scales while allowing for graft packing, vascularization, and osseointegration [107,108,109,110,111,112,113]. Compared with solid implants, architected lattices reduce implant–bone stiffness mismatch (mitigating stress shielding), create high surface area with fully interconnected porosity for tissue ingrowth, and can be contoured to patient anatomy from CT-derived geometry [23,26,56,73,104,114].

The lattice architectures used in femoral and tibial reconstruction can be grouped into several broad families, such as strut-based networks (e.g., Kelvin, Rhombic Dodecahedron, Octet, Honeycomb, Diamond), which offer adjustable stretch versus bending-dominated mechanics [21], where beam thickness and pore size control RD and stiffness; these networks are straightforward to parametrize for AM and are widely used for elastic modulus matching in bone-mimicking lattices [106,107,115,116]. There are also Triply Periodic Minimal Surface (TPMS) lattices (Gyroid, Primitive, Diamond, in sheet or skeletal form), which provide smooth, continuous surfaces with high specific strength, tunable permeability, and bone-like mean curvature, reducing notch sensitivity and facilitating powder evacuation [105,106,108,117]. Primitive-type TPMS lattices typically show higher permeability, whereas Gyroid structures often provide higher specific strength; both can be tuned to match trabecular and cortical stiffness ranges by adjusting porosity and strut or sheet thickness [115,118,119]. Stochastic and Voronoi architectures mimic the complexity and near-isotropy of trabecular bone, offer high permeability, and are frequently employed around graft windows or homogenized in finite element (FE) models to accelerate design iterations [109,112,113], Topology-optimized shells with lattice windows (often termed surface-lattice or mesh-window implants) retain an anatomic shell to support fixation and joint/contact geometry, while opening large fenestrations that host lattice infill and contain morselized graft, thereby reducing weight and increasing biological surface area [26,114,120,121]. Functionally graded lattices (FGLs) introduce spatial gradients in unit cell size, relative density, or even material composition to follow bone-stiffness distributions, concentrate strength at junctions or interfaces, and achieve more physiological load transfer; patient-specific grading can be derived directly from CT-based stiffness maps or defect reconstructions [105,108,112]. Hybrid and interpenetrating concepts include metallic lattice shells purposely designed to be graft-filled intra-operatively (often combined with biologics such as BMPs or local antibiotics) and fully bioresorbable medical-grade Poly(ϵ-Caprolactone)–Tricalcium Phosphate (mPCL–TCP) lattices with large (>70–80%) pores that underpin SGBR strategies [110,122,123,124].

**Figure 4:**
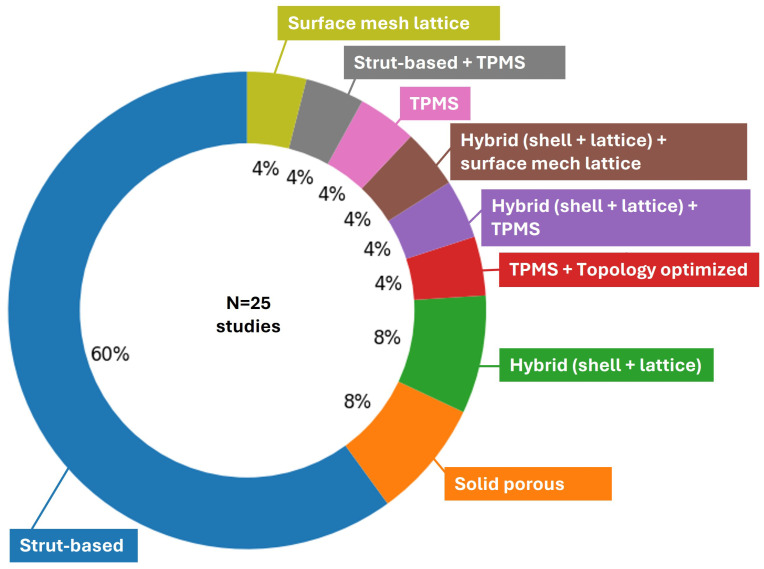
Distribution of lattice families in femoral/tibial scaffolds across selected studies [17,18,21,22,24,26,27,53,54,55,56,57,58,59,60,61,62,63,84,85,86,87,88,89,90].

Collectively, these lattice families are well-suited to femoral and tibial applications because they enable targeting of the apparent modulus into cortical and trabecular ranges; meanwhile, they maintain adequate strength, provide permeability and surface area for tissue ingrowth and graft perfusion, increase interface friction and roughness (particularly with high-porosity tantalum like architectures) for immediate stability, and are manufacturable by Laser Powder Bed Fusion (LPBF) and Electron Beam Melting (EBM) at clinically relevant feature sizes. In addition, geometry-smoothing strategies (e.g., filleting, sheet-based TPMS implementations) are increasingly used to mitigate fatigue-critical stress concentrations, thereby supporting the long-term durability required for load-bearing segmental reconstructions. As summarized in the circular plot in Figure 4, most femoral and tibial scaffolds in the selected studies are focused on strut-based architectures (15/25 studies, 60%). The remaining studies are distributed across smaller categories, including solid, non-porous constructs (2/25, 8%) and hybrid shell–lattice designs (2/25, 8%). Architectures that combine multiple design families are uncommon and appear only as single-study cases (each 1/25, 4%), including TPMS and topology-optimized, TPMS and hybrid shell–lattice, hybrid shell–lattice and surface/mesh lattice, and strut-based and TPMS. Pure TPMS-only and surface/mesh-only designs are also rare (1/25 each, 4%). Overall, the distribution indicates that long bone reconstructions still predominantly rely on conventional strut-based lattices, while TPMS-based, hybrid, and topology-informed concepts remain comparatively underrepresented and are only beginning to emerge in femoral and tibial applications.

To complement the count-based overview, Figure 4 and Figure S4 (Supplementary Materials) provide a semi-quantitative comparison of the dominant lattice families across stiffness-related mechanics, fatigue/durability reporting, transport-relevant morphology (porosity/permeability), manufacturability, feature-size constraints, and design controllability, based on parameters explicitly reported in the included studies.

The distribution of architectures in Figure 4 has implications for both design practice and the interpretation of results across studies. The dominance of strut-based lattices suggests that much of the current design space is shaped by architectures that are easier to parameterize, manufacture, and evaluate, which helps explain why they remain the most common choice in load-bearing reconstructions. At the same time, the limited representation of TPMS, hybrid shell–lattice designs, and topology-informed concepts suggests that the field has not yet established clear selection rules linking lattice family to clinical objective, such as early stability, long-term durability, or improved transport for graft integration. Therefore, comparisons across studies are most informative when the lattice family is treated as a design decision with an explicit rationale, and when the chosen architecture is discussed alongside intended mechanical function, expected failure mode, and manufacturing constraints. Altogether, the observed distribution supports clearer reporting of why a specific lattice family was selected and which performance trade-offs it is intended to address, enabling progress from cataloging architectures toward transferable design principles.

## 6. Materials and Additive Manufacturing

**Figure 5:**
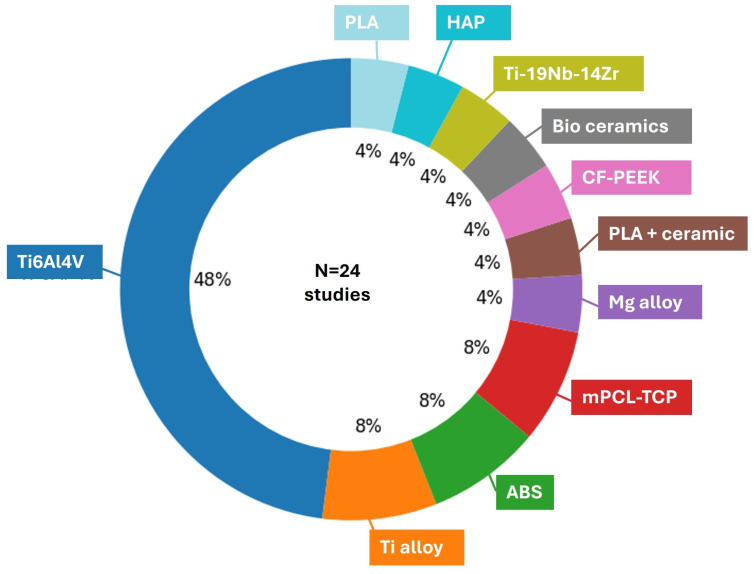
Distribution of materials in AM-based femoral/tibial scaffolds across selected studies [17,18,21,22,24,26,27,53,54,55,56,57,58,59,60,61,62,63,84,86,87,88,89,90].

**Figure 6:**
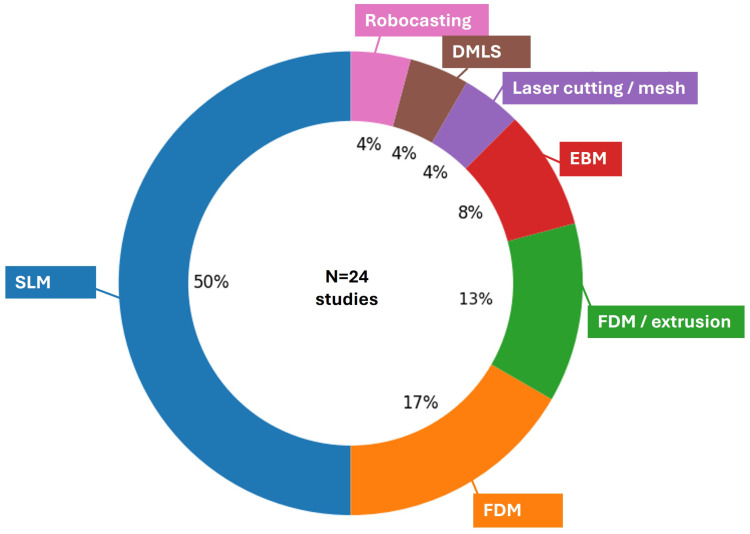
Distribution of AM methods across selected studies [17,18,21,22,24,26,27,53,54,55,56,57,58,59,60,61,62,63,84,86,87,88,89,90].

A wide range of initial biomaterials were studied for printing, Ti-based alloys, mainly Ti6Al4V (ELI with extra low O, N, C content) being the main material of interest [17,26,53,56,57,58,61,62,84,87], occasionally accompanied by pure Ti [62] or other Ti-based alloys [59,125]. Ti alloys were mainly produced using LPBF; often reported as Selectice Laser Melting (SLM) or Direct Metal Laser Sintering (DMLS) in the included studies) and EBM techniques [126,127,128]. The distribution of materials used in the AM-based femoral/tibial scaffolds is summarized in Figure 5, showing that Ti6Al4V is the most frequently reported material (12/24 studies, 48%), with smaller contributions from other titanium alloys, polymers, bioceramics [129], and resorbable composites. Figure 6 presents the distribution of manufacturing routes, where LPBF is the frequently reported process, with 48%, while Fused Deposition Modeling (FDM)-type extrusion, EBM, and more specialized methods such as DMLS, robocasting, or mesh-based fabrication appear in only a minority of reports. The AM material and process distributions (Figure 5 and Figure 6) are based on the subset of studies that explicitly report a manufacturable scaffold material and fabrication route (n = 24). Design- and simulation-based studies without an AM build ([85]) were therefore excluded from these distributions.

**Figure 7:**
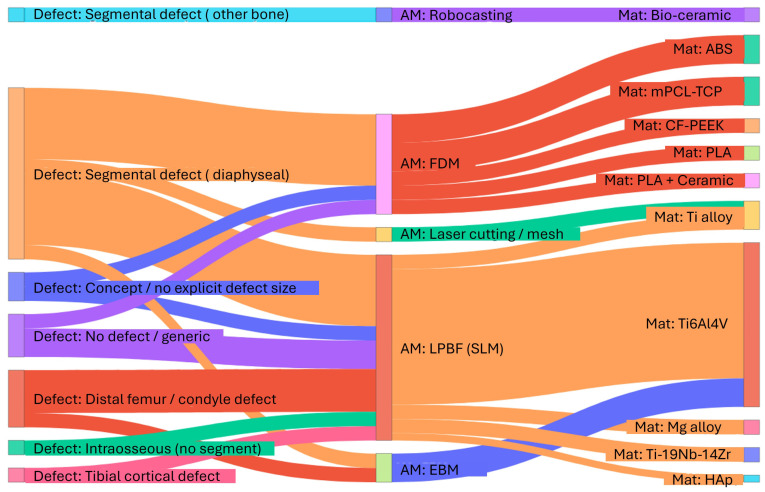
Sankey diagram linking defect type, AM method, and material in AM-based femoral/tibial scaffold studies [17,18,21,22,24,26,27,53,54,55,56,57,58,59,60,61,62,63,84,85,86,87,88,89,90].

Figure 5 and Figure 6 summarize the overall distributions of materials and AM routes across the included studies; however, these marginal distributions do not reveal how material choice and manufacturing route co-occur within specific defect contexts. To explicitly capture these associations, Figure 7 presents a Sankey diagram linking defect category, AM method, and material(apears as “mat” in the figure) for the studies included in the distribution analysis. The visualization shows a clear concentration of Ti6Al4V produced by LPBF/EBM in segmental and distal-femur reconstructions. In contrast, FDM/extrusion-based processes are more commonly paired with polymer/ceramic materials in non-segmental or concept-oriented investigations.

The material and AM distributions in Figure 5 and Figure 6, together with the associations illustrated in Figure 7, provide context for why reported performance cannot be separated from manufacturing constraints and post-processing steps. The concentration of Ti6Al4V produced by powder bed fusion indicates that much of the current evidence is anchored in a material and process pair that supports high geometric fidelity and clinically relevant strength, while also having sensitivities to surface condition, internal defects, and feature size limits that can influence fatigue behavior and osseointegration. In contrast, the smaller set of polymer, ceramic, and extrusion-based studies often reflects different design goals and should not be interpreted as equivalent to metallic reconstructions with respect to load-bearing demands or longevity expectations. Accordingly, future design decisions should treat material choice and manufacturing route as joint determinants of feasible lattice morphology, surface characteristics, and mechanical reliability. More consistent reporting of manufacturing parameters and post-processing steps would also improve interpretability and reproducibility across studies. In this sense, the distributions are not only descriptive, but also indicate that translation depends on aligning clinical function with a manufacturable architecture and a reliable process chain.

## 7. Methodological Overviews: Simulation and Experimental Studies

To complement the anatomy-, lattice-, and manufacturing-focused summaries presented above, this section reorganizes the same body of work by methodological category. Rather than repeating details on defect location, scaffold architecture, material choice, or personalization workflow, Table 3 provides a concise summary classification for each included paper by combining a study setting label with a set of method flags. The study setting label distinguishes clinical reports in humans (C), preclinical in vivo studies in animal defect/implantation models (P-vivo), and preclinical in vitro or ex vivo studies such as bench mechanical testing, cell/tissue assays, surrogate constructs, or cadaveric experiments (P-vitro/exvivo). A small subset of papers is limited to design/CAD workflows without mechanical or biological outcome data and is marked as such in Table 3; when the setting cannot be determined from the available reporting, (-) is used.

**Figure 8:**
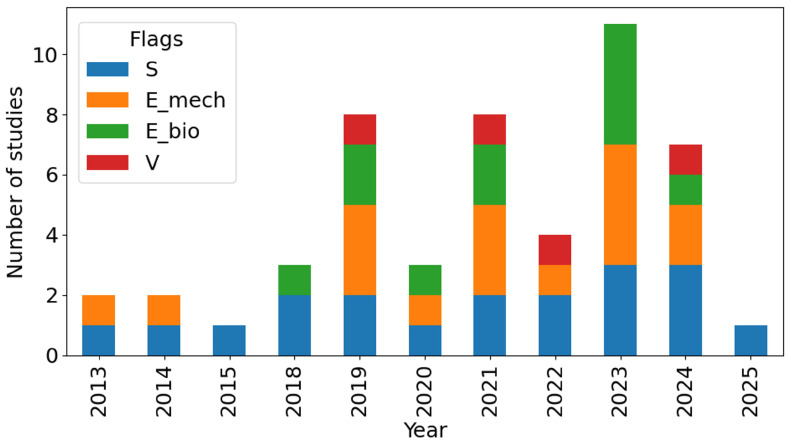
Year-related distribution of reported methods across the included studies. Stacked bars indicate the number of papers reporting simulation (S), mechanical testing (E_mech), biological or clinical endpoints (E_bio), and quantitative model measurement comparison (V); a single study may contribute to multiple categories [17,18,21,22,24,26,27,53,54,55,56,57,58,59,60,61,62,63,84,86,87,88,89,90].

Method flags summarize what each paper reports, such as simulation or numerical modeling (S, including finite element analysis), mechanical testing (E_mech), biological or clinical endpoints (E_bio), and explicit quantitative comparison between model outputs and measured results (V). Here, S does not imply a simulation-only study; it simply indicates that numerical modeling is reported and may appear alone or alongside experiments. E_mech includes bench-top or ex vivo tests such as quasi-static or cyclic loading, bending, or torsion. E_bio is used when biological or clinical outcomes are reported (e.g., in vivo healing/regeneration readouts, histology, micro-CT morphometry, bone implant contact, or patient follow-up outcomes). V is reserved for studies that provide an explicit quantitative model–measurement comparison (e.g., predicted versus measured strain, stiffness, load-sharing, or construct motion). Because many papers use hybrid pipelines, studies often carry combinations of flags (e.g., S + E_mech or S + E_mech + E_bio + V). Figure 8 summarizes the distribution of method flags across the included studies and highlights the predominance of simulation and mechanical testing over biologically anchored and quantitatively validated work.

### 7.1. Simulation and Numerical Modeling Overviews

FE simulation is a primary tool for analyzing load transfer, fixation mechanics, and failure risk in long bones, particularly the femur and tibia. Typical models range from idealized cortical–trabecular shafts and defect surrogates to fully patient-specific geometries built from CT data [56,63,84,85,90]. Depending on the study question, analyses cover axial compression, bending, and torsion, or predict stress and strain under physiological loading, single-leg stance, and gait-inspired loading, with joint reaction forces and major muscle groups applied at the hip or knee and constraints imposed at the distal femur or proximal/distal tibia [26,27,84,85]. Whole-femur models with varying elastic properties—based on Hounsfield Units (HUs)—are also used to study intramedullary nails, plates, and implants during activities such as single-leg stance or gait [85,92]. With regard to broader discussions regarding modeling challenges specific to patients, additional insights can be found in [95]. In segmental defect reconstructions, simulations are used to compare scaffold architectures, evaluate plate or nail configurations, and quantify stress shielding, micromotion, and fatigue-sensitive regions in both implant and host bone [23,26,27,58]. More advanced frameworks incorporate CT-based density–elasticity mapping, regionally graded materials, or mechanobiological criteria to guide stiffness tuning and, in some cases, predict tissue differentiation trends over time [56,58,90]. Together, these approaches define a commonly used toolbox for virtual testing of long bone reconstructions before bench-top or in vivo follow-up.

Early contributions include distal-femur scaffold–plate constructs evaluated under physiological hip and muscle loads [84], boundary-condition sensitivity analyses for retrograde intramedullary nailing [85], and mechanobiologically guided plate–scaffold optimization in ovine tibial defects [58]. More recent work extends from unit-cell and scaffold-level TPMS comparisons [22,23] to CT-based stiffness-mapped femur segments with regionally tailored porous implants [56], topology-optimized or lattice-based distal femur reconstructions [26,27], and large patient-specific femoral defects with alternative scaffold–plate–screw constructs [90]. Across these models, key differences lie in geometric complexity (lattice-only versus full bone implant constructs), material representation (homogeneous versus CT-derived density-mapped; linear elastic versus more advanced constitutive choices), and boundary/loading conditions (simplified uniaxial compression versus multi-axial, gait-inspired physiological loading).

Across the included literature, FE modeling is used mainly as a comparative tool to screen long bone reconstruction concepts before (or alongside) bench-top and preclinical follow-up. Most studies build femur/tibia constructs with simplified, predominantly linear-elastic material descriptions and then evaluate how implant architecture and fixation strategy shape load transfer, stress/strain distributions, construct stiffness, and interfragmentary motion under idealized representations of weight-bearing (often single-leg-stance or gait-inspired loading with distal constraints) [23,24,26,53,58,84,85,90]. While the exact boundary conditions and interface definitions differ substantially across papers and can meaningfully shift predicted stress patterns and stiffness trends [85,92], the FE outputs are consistently used to articulate design trade-offs, most commonly the balance between porosity/permeability and mechanical stability [23,54,84], or between stiffness matching and stress shielding [24,56,58]. Importantly, several studies go beyond simulation-only reporting by calibrating material parameters from mechanical characterization or by quantitatively checking model predictions against measured stiffness/strain fields in scaffold blocks or construct-level tests [22,26,27,55,56,57,63,88]. Consequently, we view FE results mainly as proof of comparative positioning and understanding of mechanisms, while the study-specific modeling choices, simplifications, and validation status are documented in detail in Part 2.

### 7.2. Experimental Overview: Mechanical Testing

In experimental settings, reconstructive assessments of long bones are conducted either using individual scaffold samples or by utilizing bone implant constructs set up in surrogate or cadaveric segments. Bench-top characterization of printed architectures commonly begins with uniaxial compression (and, less frequently, indentation or combined loading) to quantify apparent stiffness, strength, and energy absorption and to assist with material calibration for future construct-related analyses [22,55,56,84,88]. In the context of constructs, numerous studies attach defected femur segments (either synthetic or cadaveric) to plate-scaffold or implant systems, using quasi-static axial compression or a combination of compression and bending to simulate weight-bearing conditions. Common measurements taken from these studies include overall construct stiffness, load distribution, and the relative movement at the defect site [26,55,61]. Several research studies broaden the mechanical assessment beyond simple loading by including cyclic testing or torsional endpoints to examine durability and stability in rotation, especially during preclinical evaluations post-healing [62,87]. In total, these experiments offer significant evaluations of feasibility and comparative stability; however, they are frequently conducted under simplified boundary conditions and restricted loading scenarios, which may not fully capture the multi-directional and fatigue-driven requirements of everyday activities. In addition to composite-bone and cadaveric models, preclinical research provides biological data while primarily depending on regulated or simplified mechanical conditions. In vivo animal models link implant architecture and fixation strategy to bone formation and incorporation as measured by radiography, micro-CT, histology, and, in some cases, ex vivo mechanical testing of explanted segments [18,27,59,86,87]. Alternative surrogate methods, such as femur substitutes made with FDM printing and associated constructs, have been employed to investigate plate fixation and to establish consistent testing environments for evaluating mechanical responses among different designs [60,63]. Finally, a small number of papers primarily emphasize planning or geometry-generation workflows (design/CAD), rather than reporting new mechanical or biological outcome data [89].

### 7.3. Limitations of the Study Base

Across the included studies, the strength of conclusions depends strongly on the study setting and test conditions. Mechanical testing provides direct assessments of the stiffness and failure characteristics of constructs; however, it often relies on simplified loading conditions, brief testing durations, and surrogate substrates or fixtures that do not represent the full range of multi-directional and fatigue loading experienced in everyday life. Preclinical in vivo studies provide insights into biological responses and bone ingrowth; however, results can vary significantly with species, defect size, fixation technique, and duration of follow-up. Hence, it is important to exercise caution when applying these findings to long bone reconstruction in adult humans. Clinical reports provide the most direct demonstration of feasibility in real indications, but they remain limited in number and are commonly small series without comparative arms. In general, simulation and bench-top testing dominate the study base, whereas longer follow-up, fatigue-oriented protocols, larger clinical cohorts, and consistent quantitative model-to-measurement comparisons remain relatively uncommon.

Taken together, Table 3 and Figure 8 summarize how the included papers map onto simulation (S, mechanical testing (E_mech), biological or clinical endpoints (E_bio), and explicit quantitative model–measurement comparison (V). The distribution shows that the literature is dominated by numerical studies and bench-top mechanical testing, while fewer studies report biological follow-up or quantitative validation, indicating that long bone lattice reconstructions are still primarily supported by in silico and experimental bench evidence. This has two implications for synthesis and decision making: first, findings from simulation-focused studies or short-duration mechanical tests should be interpreted as comparative guidance for design selection and risk identification rather than direct predictors of clinical performance; second, the limited linkage between model outputs, measured construct behavior, and biological incorporation underscores the need for tighter integration of modeling, mechanical testing, and follow-up endpoints within a unified workflow. In the subsections that follow (and in the companion clinical review Part 2), this coding is used to contrast modeling assumptions and experimental setups and to highlight recurring gaps, including simplified loading, limited consideration of time-dependent remodeling and fatigue, sparse biological follow-up, and the near-absence of systematic uncertainty quantification.

## 8. Challenges and Future Directions

Despite significant advancements in CT-related, patient-specific designs and modular porous reconstruction, there are still challenges to address: clinical and defect-centered context, fixation and mechanical environment, anatomical/fixation registries, lattice architecture, materials and AM, and methodological pipelines. Addressing these gaps is essential if architected scaffolds are to move from technically impressive case series to reproducible treatment strategies.

From a clinical viewpoint, there is a growing evidence base supporting these approaches. Defect-centered descriptors (size, location, morphology, host biology, infection status) are inconsistently reported, which limits meta-analysis and complicates indication setting across methods. Follow-up periods are often brief, and long-term survival data for large segmental defects reconstructed with porous scaffolds is still sparse in comparison with more established options such as bone transport or megaprostheses. Future work should focus on defect-stratified registries with harmonized classifications, imaging protocols, and both functional and patient-reported outcomes, ideally spanning multiple centers and reconstruction strategies. Such datasets are needed to define safe defect-size limits for different implant concepts, quantify complication patterns in high-risk subgroups, and support evidence-based guidelines rather than expert opinion.

Fixation remains a major source of variability. Across clinical, preclinical, and in silico studies, plates, nails, external fixators, hybrid constructs, and scaffold-integrated fixation are used with very different stiffness profiles and boundary conditions, often without systematic comparison. Short-term stability is usually documented, but the longer-term evolution of construct stiffness, stress shielding, and periprosthetic bone quality is rarely followed in a standardized way. Future work should treat fixation strategy, scaffold design, and rehabilitation protocol as a coupled system: construct-level optimization that targets strain windows conducive to regeneration, explicit reporting of weight-bearing regimes, and, where possible, mechanical monitoring (e.g., instrumented fixators or serial stiffness measurements). In parallel, more robust fatigue and damage-tolerance data are needed for both scaffolds and fixation hardware in the specific loading environments created by long bone defects.

The anatomical and fixation registry in this review highlights how strongly outcomes depend on defect archetype and host-bone context, but it also exposes gaps. Certain clinically important scenarios—very long diaphyseal defects, multi-segment meta-diaphyseal defects, periarticular bone loss combined with ligament insufficiency—are still represented mainly by isolated reports or purely computational models. Tibial defects, pediatric cases, and osteoporotic hosts are under-represented compared with adult femoral diaphyseal models. A key future direction is to build structured datasets that span age groups, bone segments, and etiologies, capturing not only implant and fixation choices but also time-resolved imaging, biological adjuncts, and reoperation pathways. Such datasets could underpin decision-support tools that suggest construct families and scaffold templates tailored to the defect pattern and host-specific factors.

On the design side, only a small portion of the available lattice and gradient design space has been explored in long bone reconstruction. Most studies rely on a limited set of unit-cell families and relatively simple grading patterns, typically tuned for apparent stiffness and, to a lesser extent, permeability. Multiscale, hierarchical architectures that couple cortical-scale load paths with trabecular-like interiors, or that combine different lattice families within a single implant, are largely conceptual rather than validated. There is also little quantitative guidance on trade-offs between mechanical robustness, permeability, surface area for graft and ingrowth, and manufacturability. Future research should therefore develop systematic design maps that integrate experiments, FEA, and mechanobiological models to relate defect type and fixation scheme to recommended lattice families, porosity ranges, gradients, and graft windows. Optimization-based frameworks that can propose and rank architectures subject to clinical and manufacturing constraints are a natural next step.

Materials and AM processes pose their own challenges. For metallic lattices, fatigue behavior under mixed mode loading, the effects of surface condition and post-processing on long-term performance, and the consequences of wear/corrosion products in large surface-area constructs are all incompletely characterized. Polymer–ceramic and bioresorbable lattices, while attractive for younger patients and SGBR concepts, pose open questions regarding degradation kinetics, evolving mechanical properties, and the interplay among resorption, fixation, and bone formation in weight-bearing segments. Across all materials, there is a need for robust, clinically compatible QA frameworks that link AM process parameters and in-process monitoring to mechanical and permeability specifications at the scaffold scale. Future work should therefore focus on defining tolerances for pore size, connectivity, and surface roughness; integrating non-destructive evaluation into AM workflows; and developing mixed-material strategies (e.g., Ti shells with resorbable cores) that are amenable to regulatory approval.

Methodologically, in silico and bench-top pipelines are powerful but still far from harmonized. FE models differ markedly in segmentation practice, density elasticity mapping, material laws, and boundary conditions, which makes it hard to compare results across studies or reuse them in regulatory contexts. Experimental work often focuses on quasi-static tests of isolated scaffolds or simplified constructs, with relatively little attention to fatigue, multi-axial loading, or time-dependent remodeling. Biological data (both in vitro and in vivo) are likewise heterogeneous in species, defect models, and outcome metrics.

Looking ahead, important steps including agreeing on shared benchmark geometries and loading scenarios for long bone defects; defining minimal reporting standards for FE modeling and mechanical testing; routinely incorporating uncertainty quantification and sensitivity analysis into design studies; and linking mechanobiological models more closely with longitudinal preclinical data to move from short-term mechanical surrogates toward true healing outcomes. In the longer term, virtual cohorts and in silico trials that combine patient CT libraries with validated FE pipelines could help de-risk new scaffold concepts before they reach the clinic.

Finally, current imaging-to-implant workflows remain labor-intensive and center-specific. Segmentation, lattice design, construct optimization, and print preparation are typically executed across multiple software platforms by expert users, with case-by-case regulatory pathways and informal QA. This limits scalability and makes it hard to reproduce promising workflows in other hospitals. Future work should aim for interoperable Picture Archiving and Communication System (PACS)–planning–FEM–AM pipelines with embedded AI tools for segmentation, landmarking, and automatic proposal of implant/fixation templates; semi-automatic verification against predefined mechanical and anatomical constraints; and structured export of design and validation data for regulatory submission. Such pipelines would compress design time, reduce operator dependence, and make sophisticated lattice and mixed-material concepts accessible beyond a few specialized centers.

To summarize how defect descriptors (size, morphology, infection status) and host factors (bone quality) map to commonly reported reconstruction choices (implant architecture, fixation concept, and material/AM route), we provide a conceptual decision tree in Figure S5 (Supplementary Materials). This schematic is intended as a synthesis aid rather than a prescriptive clinical algorithm.

Taken together, these challenges define a clear translational agenda: build richer, defect-centered clinical datasets; co-optimize scaffold, fixation, and rehabilitation; systematically explore the lattice and material design space; standardize modeling and testing; and industrialize the imaging-to-implant workflow. If these elements can be aligned, patient-specific and modular porous constructs are well positioned to become a routine option for segmental femoral and tibial reconstruction rather than a niche solution for exceptional cases.

## 9. Conclusions and Outlook

This review shows how CT-based, patient-specific reconstruction with architected porous implants is reshaping treatment options for large segmental defects in long bones. When the available studies are viewed together from purely computational work through bench-top mechanical and biological tests, preclinical animal models, and early clinical series, a common pattern emerges: successful reconstruction does not hinge on a single “ideal” scaffold, but on achieving a coherent match between defect archetype, fixation strategy, lattice architecture, and host biology. When that match is achieved, porous metallic and polymer–ceramic constructs can provide stable load transfer, space for bone regeneration, and restoration of limb alignment in situations that previously often led to amputation or high-morbidity salvage procedures.

At the same time, the field is limited by dispersed reporting and a lack of consistent detail on defect geometry, fixation configuration, loading conditions, and healing outcomes. Across the 27 included studies, simulation and mechanical testing dominate (70% and 56%, respectively), while in vivo biological endpoints are reported in only 33%, underscoring a persistent validation gap that limits translation. Closing this gap will require three complementary developments: defect-centered datasets that consistently capture anatomy, mechanics, biology, and longitudinal outcomes; design frameworks that explicitly couple scaffold architecture and fixation to the mechanical environment of healing; and robust, standardized workflows from imaging to implant, including quality assurance and validation of both simulations and experiments. If these elements can be integrated and shared more widely, patient-specific porous reconstructions have the potential to move from exceptional case reports to reproducible, standard of care workflows for segmental femoral and tibial defects.

## Figures and Tables

**Table 1:** Quantitative mechanobiology extracted from selected studies; each row states the reported metric(s), the magnitude(s), the biological interpretation used by the authors, and the primary design lever(s) discussed.

Year, Ref	Metric Reported	Magnitude	Interpretation + Design Lever
2014, [61]	Porosity/geometry with stiffness gradient	Pore 500 μm; struts 120/170/230 μm; porosity 84.22/77.68/68.45%; modulus 0.55/1.40/3.49 GPa	Stiffness-dependent load transfer trend (qualitative in text), supports stiffness-matching rationale.
2018, [58]	Plate stiffness; scaffold stiffness; pore-level strains; FE loads	Plate: 555 vs. 2857 N/mm; scaffold: 0.20–3.09 kN/mm; strains: 0.23–0.6% (autograft pores), 0.65–1.3% (callus pores); cited formation range 5% to 0.04%, resorption below ∼0.04%; FE: 1372 N compression, 86 N bending	Directly links fixation stiffness to strain environment (load sharing vs stress shielding) and mechanobiology-relevant strain windows.
2018, [54]	Defect-/construct displacement (mm)	0.0031–0.012 mm; intact 0.002 mm	Used to discuss relative motion and stress shielding reduction across porosity designs.
2019, [60]	Porosity/strength/modulus ranges	Porosity up to 82.7%; strength 1.76–9.34 MPa; modulus 52.2–212 MPa	Context-only mechanical ranges; stress shielding mentioned generally (no micromotion/strain magnitudes).
2019, [55]	Load sharing + strain energy framing	Load transfer described qualitatively (no % load share or stiffness reported	Rigid plate: majority load through plate, limited through scaffold (stress shielding risk); high strain energy: excessive motion beyond physiological limits; balance stability vs load transfer.
2019, [17]	Clinical loading protocol timing	PWB max 35 kg for 6 weeks; progress to FWB over next 6 weeks	Supports time-varying mechanical environment via rehabilitation protocol (no micromotion/strain magnitudes).
2020, [53]	Interface strain (mechanostat-style)	Damage strain threshold ∼4000 με	Above threshold: microdamage risk, delayed union/nonunion; below: favorable early-stage indicator; note scale/modeling limitations.
2021, [57]	Design geometry; FE peak stress; qualitative micromotion/time	Pore 400–600 μm; strut 240–320 μm; porosity 60–80%; peak stress 12.09 MPa; 1000 N, 3.09 MPa; 2000 N, 37.21 MPa; 3000 N	Micromotion/stress stimulation invoked but not quantified; micromotion decreases and disappears over time; plate-side stress shielding noted; porous structure mitigates shielding.
2021, [56]	Stress shielding framed via strain distribution	No numeric magnitude reported	Stress shielding as strain reduction; motivates stiffness distribution matching toward intact-like strain profile (remodeling-relevant).
2022, [26]	Time dependence: ingrowth/remodeling	Time-dependent effect described (no µm/mm/time curve reported)	Bone ingrowth/remodeling increases stability; long-term stable implant–bone complex.
2023, [24]	Interface relative displacement; strain window	150 μm critical displacement; 1–5% strain (incl. ∼1% early-stage note)	>150 μm: fibrous risk; 1–5% presented as conducive in that porous scaffold context; emphasizes stability + stiffness mismatch (stress shielding) framing.
2023, [18]	Fixator stiffness; load sharing evolution; remodeling timing	$K_{f}$ = 593 N/mm; >90% force through fixator (first fortnight); reversal after ∼30 days; callus stiffness ∼650 N/mm; day 40 to ∼7 kN/mm ∼20 days later; remodeling transition ∼200 days	Quantifies early stability—load transfer—remodeling phases; provides time-resolved mechanobiology.
2023, [59]	Imaging-based interface proxies; stiffness mismatch	10 μm reference limit; voxel/pixel 6.8 μm; BII = 3.3 μm; BIC = 95.4%; cortical bone 25–30 GPa; titanium alloy 38 GPa	Quantifies interface quality (proxy for stability) and relates stiffness mismatch to stress concentration considerations.
2023, [62]	Geometry + loading/FE (no micromotion)	8 × 8 × 5.8 mm; struts 200–500 μm; porosity 50% vs 62%; cyclic 150 N; FE pressure 1.25 MPa	Stress shielding/modulus mismatch framing without reporting micromotion/gap motion/strain thresholds.
2023, [63]	Strain-to-failure (model)	Fracture signs at 1.9%; failure 208.58 MPa, strain 1.99%	Not a mechanoregulation/ingrowth paper; can be used only as bounded strain magnitude context.
2024, [27]	Bone strain target + validation; design sensitivity	Max bone strain ≤ 4000 με; conducive strains ∼2046–2253 με; examples: 1752.6 vs. 1687.9 με, 2046.4 vs. 2133.7 με, 2252.57 vs. 2292.55 με; pillar dia 0.9 mm → modulus 14.99–15.88 GPa and strain >4000 με; bone cement modulus 2.65 GPa; intraluminal growth >79.8%	Uses ∼4000 με as unfavorable upper bound; ties geometry/material choices to stress shielding/osseointegration outcomes.

**Table 2:** Overview of anatomical site, defect archetype, study model, imaging pipeline, fixation class, and translational stage across the included long bone defect studies.

Ref, Year	Site	Defect Archetype	Model Type	Imaging Pipeline	Fixation Class	Readiness
2013, [84]	Femur distal diaphysis	Segmental, 30 mm	Bench + FE	CT with HU mapping	Lateral locking plate	Bench and in silico
2014, [61]	Femur mid diaphysis	Segmental, 6 mm	Ex vivo (rat femurs)	Micro CT (implant architecture)	Internal PEEK locking plate (RatFix)	Bench
2015, [85]	Femur distal diaphysis	Osteotomy, 5 mm	FE only	No DICOM (public model)	Retrograde IM nail + interlocks	In silico
2018, [58], Case 1	Tibia mid diaphysis	Critical size segmental, 4 cm	In vivo (large animal)	Radiographs + endpoint imaging	Plate fixation (two variants)	Preclinical in vivo
2018, [58], Case 2	Femur and humerus (clinical sites)	Large segmental defects	Clinical cohort	CT planning + follow up imaging	Plate constructs with Ti mesh scaffold	Clinical
2018, [54]	Femur diaphysis	Segmental (model limited), 8 mm	FE and concept validation	CT segmentation (Mimics)	No fixation hardware	In silico
2019, [55]	Femur mid shaft (composite)	Segmental cylinder filled with scaffold	Bench + FE	Optical scan + DIC	Lateral condylar plate	Bench and in silico
2019, [22]	Generic femur segment (not specified)	No defect (material study)	Bench (in vitro)	No CT (SEM used)	No fixation hardware	Bench
2019, [60]	Femur mid diaphysis	Segmental model (no pathology)	Bench (fabrication tests)	CT segmentation (Mimics)	No fixation hardware	Bench
2019, [17], (Patient A)	Femur diaphysis	Segmental, 15.2 cm	Clinical case	CT planning + radiographs + follow up CT	Ti cage + IM nail (Masquelet)	Clinical
2019, [17], (Patient B)	Femur metadiaphysis	Segmental, 15.1 cm	Clinical case	CT planning + radiographs + follow up CT	Ti cage + lateral locked plate	Clinical
2019, [17], (Patient C)	Femur metadiaphysis	Segmental, 18.4 cm	Clinical case	CT planning + radiographs + follow up CT	Ti cage + lateral locked plate	Clinical
2019, [17], (Patient D)	Femur metadiaphysis	Segmental, 10.3 cm	Clinical case	CT planning + radiographs + follow up CT	Ti cage + lateral locked plate	Clinical
[17], 2019, (Patient E)	Femur diaphysis	Segmental, 11.1 cm	Clinical case	CT planning + radiographs + follow up CT	Ti cage + IM nail	Clinical
2020, [86]	Femur mid diaphysis (rat)	Segmental, 3 mm	In vivo (rat)	X ray + micro CT	PEEK locking plate (RatFix)	Preclinical in vivo
2020, [53]	Femur distal (lateral condyle region)	Large segmental defect (trauma)	Surgical planning case	CT-based reconstruction	PSI implant + lateral locking plate	In silico
2021, [56]	Femur (whole model sections)	Resection replacement concept	Bench + FE	CT segmentation (Mimics)	No fixation hardware (bonded in FE)	Bench and in silico
2021, [87]	Femur diaphysis (rat)	Critical size segmental, 8 mm	In vivo (rat)	X ray + micro CT	Plate fixation across defect	Preclinical in vivo
2012, [21]	Generic femur model	No explicit defect (scaffold study)	Bench + FE	CT-based modeling pipeline	No fixation hardware	Bench and in silico
2021, [57], Case 1	Femur diaphysis (human)	Segmental, 11 cm	Clinical case	Radiographs (CT not detailed)	Patient-specific implant + IM nail	Clinical
2021, [57], Case 2	Femur mid diaphysis (sheep)	Critical size segmental, 4 cm	In vivo (large animal)	Radiographs + micro CT + FE	Plate and screws integrated with implant	Preclinical in vivo
2021, [57], Case 3	Femur mid diaphysis (sheep)	Critical size segmental, 4 cm	In vivo (large animal)	Micro CT-based quantification	Plate and screws integrated with implant	Preclinical in vivo
2022, [23]	Femur (model defined)	Critical size segmental, 50 mm	FE only	CT segmentation	No fixation hardware	In silico
2022, [26]	Distal lateral femur	Large defect (anatomy defined)	Bench + FE (composite)	Patient CT for sizing (details not reported)	Lateral locking plate + screws	Bench and in silico
2023, [24]	Femur (intercalary concept)	Large segmental defect (not numeric)	FE only	CT segmentation (Mimics, Magics)	Integrated fixation device (personalized)	In silico
2023, [62]	Concept (multiple sites illustrated)	Modular blocks (no single defect)	Bench (in vitro + mechanical)	Micro CT (scaffold morphology)	No fixation hardware	Bench
2023, [63]	Femur whole bone model	No defect (anatomical model)	Bench + FE	CT segmentation	No fixation hardware	Bench
2023, [18]	Metatarsus (sheep)	Segmental, 15 mm	In vivo (large animal)	Pre op CT + follow up imaging	External fixator (Ilizarov type)	Preclinical in vivo
[59], 2023	Tibia and metatarsal (sheep)	Intraosseous implants (no segmental)	In vivo (large animal)	Post explant XCT + segmentation	No fixation hardware	Preclinical in vivo
[88], 2024	Tibia mid shaft (rabbit model)	Critical size cortical defect	Prototype + FE	CT reconstruction	Curved plate + screws integrated with cage	Bench and in silico
[89], 2024	Femur shaft	Large shaft defects (two scenarios)	Prototype concept	2D medical images (X ray)	IM nail + modular blocks	Bench
[27], 2024	Distal femur	Defect model 25 mm (plus animal test)	FE + bench + in vivo	CT and post op micro CT	Lateral reconstruction plate + screws	Preclinical in vivo
[90], 2025	Distal femur	Large defect, 82 mm	FE only	CT segmentation	Scaffold + connection plates + screws	In silico

**Table 3:** Overview of methodological distribution of included studies.

Year, Ref	Evidence Tier	Flags (S, E_mech, E_bio, V)
2013, [84]	P-vitro/exvivo	S + E_mech
2014, [61]	P-vitro/exvivo	S + E_mech
2015, [85]	-	S
2018, [58]	P-vivo	S + E_bio
2018, [54]	-	S
2019, [55]	P-vitro/exvivo	S + E_mech + V
2019, [22]	P-vitro/exvivo	S + E_mech + E_bio.
2019, [60]	P-vitro/exvivo	E_mech
2019, [17]	C + P-vitro/exvivo	E_bio
2020, [86]	P-vivo (+ex vivo)	E_mech + E_bio
2020, [53]	-	S
2021, [56]	P-vitro/exvivo	S + E_mech + V
2021, [87]	P-vivo	E_mech + E_bio
2021, [57]	C (human)	E_bio
2021, [21]	P-vitro/exvivo	E_mech + S
2022, [23]	-	S
2022, [26]	P-vitro/exvivo	S + E_mech + V
2023, [24]	-	S
2023, [62]	P-vivo/P-vitro	E_mech + E_bio
2023, [63]	P-vitro/exvivo	S + E_mech
2023, [91]	C (human), P-vitro/exvivo	S + E_mech + E_bio
2023, [18]	P-vivo	E_bio, E_mech
2023, [59]	P-vivo	E_bio
2024, [88]	P-vitro/exvivo	S + E_mech
2024, [89]	(design/CAD only)	-
2024, [92]	-	S
2024, [27]	P-vivo + P-vitro/exvivo	S + E_mech + E_bio + V
2025, [90]	-	S

## Data Availability

Some or all of the data, and models that support the findings of this study, are available from the corresponding author upon request.

## Supplementary Materials

The following supporting information can be downloaded at: https://www.mdpi.com/article/10.3390/biomimetics11020128/s1.

## Abbreviations

The following abbreviations are used in this manuscript:

ABG	Autologous bone graft
ABS	Acrylonitrile–butadiene–styrene
AM	Additive manufacturing
AO	Arbeitsgemeinschaft für Osteosynthesefragen
ATS	Assemblable Titanium Scaffold
BIC	Bone–implant contact
BII	Bone–implant interface (distance)
BMP-2	Bone morphogenetic protein 2
C	Clinical evidence (clinical study)
CAD	Computer-aided design
CCD	Charge-coupled device
CF-PEEK	Carbon-fiber-reinforced polyetheretherketone
CLSM	Confocal laser scanning microscopy
CPP	Calcium phosphate phase/powder
CSBD	Critical-sized bone defect
CT	Computed tomography
DIC	Digital image correlation
DICOM	Digital imaging and communications in medicine
DMLS	Direct metal laser sintering
E_bio	In vitro biological experiments
E_mech	In vitro mechanical experiments
EBM	Electron beam melting
FDM	Fused deposition modeling
EDX	Energy-Dispersive X-ray
FEA	Finite element analyses
FEM	Finite element method/model
FGL	Functionally graded lattice
HA	Hydroxyapatite
HU	Hounsfield unit
IM	Intramedullary
LCP	Locking compression plate
LPBF	Laser powder bed fusion
mPCL–TCP	Medical-grade poly(ϵ-caprolactone)–tricalcium phosphate
ML	Machine learning
MRI	Magnetic resonance imaging
NIH	National Institutes of Health
OLS	Optimal lattice structure
ORIF	Open reduction and internal fixation
PACS	Picture archiving and communication system
PEEK	Polyetheretherketone
PSI	Patient-specific implants
PSLI	Patient-specific bone implants
PMMA	Poly(methyl methacrylate)
PROs	Patient-reported outcomes
RD	Relative density
ROM	Range of motion
S	Simulation (numerical/FEM)
SEM	Scanning electron microscopy
SHG	Second-harmonic generation
SGBR	Scaffold-guided bone regeneration
SLA	Stereolithography
SLM	Selective laser melting
SP	Semi-porous (ATS unit)
THA	Total hip arthroplasty
Ti6Al4V	Titanium alloy with 6% Al and 4% V
Ti6Al4V ELI	Extra–low interstitial Ti6Al4V
TPMS	Triply periodic minimal surface
TKA	Total knee arthroplasty
V	Validation (explicit quantitative model–measurement comparison)
XCT	X-ray computed tomography
μCT	Micro–computed tomography

## References

[1] Marrara G., Zampogna B., Schick V.D., Larizza L., Rizzo P., Sanzarello I., Nanni M., Leonetti D. (2024). Post-Traumatic Segmental Tibial Defects Management: A Systematic Review of the Literature. Appl. Sci..

[2] Wang Q., Ma T., Li Z., Zhang K., Huang Q. (2024). Semi-focal bone transport versus traditional bone transport technique for the management of large tibial bone defects after trauma. Sci. Rep..

[3] Lu Y., Lai C.Y., Lai P.J., Yu Y.H. (2023). Induced Membrane Technique for the Management of Segmental Femoral Defects: A Systematic Review and Meta-Analysis of Individual Participant Data. Orthop. Surg..

[4] Schick V.D., Zampogna B., Marrara G., Siracusano L., Larizza L., Calaciura S., Sanzarello I., Marinozzi A., Leonetti D. (2025). Custom-Made 3D-Printed Titanium Implants for Managing Segmental Distal Tibial Bone Defects: A Systematic Literature Review. J. Clin. Med..

[5] McAnena A.P., McClennen T., Zheng H. (2024). Patient-specific 3-dimensional-printed orthopedic implants and surgical devices are potential alternatives to conventional technology but require additional characterization. Clin. Orthop. Surg..

[6] Li Z., Lu M., Zhang Y., Wang J., Wang Y., Gong T., He X., Luo Y., Zhou Y., Min L. (2024). 3D-Printed personalized lattice implant as an innovative strategy to reconstruct geographic defects in load-bearing bones. Orthop. Surg..

[7] Ma J., Li Y., Mi Y., Gong Q., Zhang P., Meng B., Wang J., Wang J., Fan Y. (2024). Novel 3D printed TPMS scaffolds: Microstructure, characteristics and applications in bone regeneration. J. Tissue Eng..

[8] Wakjira Y., Cioni A., Lemu H.G. (2025). Current status of the application of additive-manufactured TPMS structure in bone tissue engineering. Prog. Addit. Manuf..

[9] Tauheed M., Alsheghri A., Dalaq A.S., Al-Rub R.K.A. (2025). Bone-inspired lattice structures for biomedical applications: Design, pore network analysis, and mechanical performance. Results Eng..

[10] Gandhi R., Salmi M., Roy B., Pagliari L., Concli F. (2025). Mechanical performance, fatigue behaviour, and biointegration of additively manufactured architected lattices. Virtual Phys. Prototyp..

[11] Alkentar R., Kladovasilakis N., Tzetzis D., Mankovits T. (2023). Effects of pore size parameters of titanium additively manufactured lattice structures on the osseointegration process in orthopedic applications: A comprehensive review. Crystals.

[12] Poelert S., Valstar E., Weinans H., Zadpoor A.A. (2013). Patient-specific finite element modeling of bones. Proc. Inst. Mech. Eng. Part H J. Eng. Med..

[13] Taylor M., Bryan R., Galloway F. (2013). Accounting for patient variability in finite element analysis of the intact and implanted hip and knee: A review. Int. J. Numer. Methods Biomed. Eng..

[14] Wu P.K., Tsai W.C., Wang H.W., Lin C.L. (2025). Establishment of design guidelines for patient-specific 3D-printed reconstruction implants for large distal femoral defects. Mater. Des..

[15] Namvar A., Lozanovski B., Downing D., Williamson T., Kastrati E., Shidid D., Hill D., Buehner U., Ryan S., Choong P.F. (2024). Finite element analysis of patient-specific additive-manufactured implants. Front. Bioeng. Biotechnol..

[16] Laubach M., Cheers G.M., Frankenbach-Désor T., Weimer L.P., Baumgartner H., Böcker W., Burgkart R., Cidonio G., D’Este M., Dirnagl U. (2025). Clinical translation of 3D-printed patient-specific bone implants: A consensus statement. Int. J. Surg..

[17] Tetsworth K., Woloszyk A., Glatt V. (2019). 3D printed titanium cages combined with the Masquelet technique for the reconstruction of segmental femoral defects: Preliminary clinical results and molecular analysis of the biological activity of human-induced membranes. OTA Int..

[18] Blázquez-Carmona P., Mora-Macías J., Martínez-Vázquez F.J., Morgaz J., Domínguez J., Reina-Romo E. (2023). Mechanics predicts effective critical-size bone regeneration using 3d-printed bioceramic scaffolds. Tissue Eng. Regen. Med..

[19] Goldstein R.Y., Jordan C.J., McLaurin T.M., Grant A. (2013). The Evolution of the Ilizarov Technique. Bull. Hosp. Jt. Dis..

[20] Gautier E., Sommer C. (2003). Guidelines for the clinical application of the LCP. Injury.

[21] Shams M., Mansurov Z., Daulbayev C., Bakbolat B. (2021). Effect of Lattice Structure and Composite Precursor on Mechanical Properties of 3D-Printed Bone Scaffolds. Eurasian-Chem.-Technol. J..

[22] Jin H., Zhuo Y., Sun Y., Fu H., Han Z. (2019). Microstructure design and degradation performance in vitro of three-dimensional printed bioscaffold for bone tissue engineering. Adv. Mech. Eng..

[23] Verma R., Kumar J., Singh N.K., Rai S.K., Saxena K.K., Xu J. (2022). Design and analysis of biomedical scaffolds using TPMS-based porous structures inspired from additive manufacturing. Coatings.

[24] Liu L., Liu C., Deng C., Wang X., Liu X., Luo M., Wang S., Liu J. (2023). Design and performance analysis of 3D-printed stiffness gradient femoral scaffold. J. Orthop. Surg. Res..

[25] Davoodi E., Montazerian H., Mirhakimi A.S., Zhianmanesh M., Ibhadode O., Shahabad S.I., Esmaeilizadeh R., Sarikhani E., Toorandaz S., Sarabi S.A. (2022). Additively manufactured metallic biomaterials. Bioact. Mater..

[26] Wu P.K., Lee C.W., Sun W.H., Lin C.L. (2021). Biomechanical analysis and design method for patient-specific reconstructive implants for large bone defects of the distal lateral femur. Biosensors.

[27] Chang C.M., Wong P.C., Ou S.L., Ko C.E., Wang Y.T. (2024). Optimizing implant lattice design for large distal femur defects: Stimulating interface bone growth to enhance osseointegration. Int. J. Bioprinting.

[28] Findeisen S., Schwilk M., Haubruck P., Ferbert T., Helbig L., Miska M., Schmidmaier G., Tanner M.C. (2023). Matched-Pair Analysis: Large-Sized Defects in Surgery of Lower Limb Nonunions. J. Clin. Med..

[29] Ma X.Y., Yuan H., Cui D., Liu B., Han T.Y., Yu H.L., Zhou D.P. (2023). Management of segmental defects post open distal femur fracture using a titanium cage combined with the Masquelet technique A single-centre report of 23 cases. Injury.

[30] Cao Z.m., Sui X.l., Xiao Y., Qing L.m., Wu P.f., Tang J.y. (2023). Efficacy comparison of vascularized iliac crest bone flap and Ilizarov bone transport in the treatment of traumatic bone defects of the tibia combined with large soft tissue defects. J. Orthop. Surg. Res..

[31] Dheenadhayalan J., Imran A., Devendra A., Venkatramani H., Velmurugesan P.S., Rajasekaran S., Sabapathy S.R. (2024). Can locking plate fixation and free Vascularised fibular transfer with skin island achieve good functional outcome in the treatment of large bone defects of Tibia? A study of 26 cases. Injury.

[32] Feng D., Zhang Y., Wu W., Jia H., Ma C. (2023). Docking site complications analysis of Ilizarov bone transport technique in the treatment of tibial bone defects. J. Orthop. Surg. Res..

[33] Vahabi A., Kaya H., Kerekulov B., Biçer A., Keçeci B., Sabah D. (2025). Does Augmenting Irradiated Autografts With Free Vascularized Fibula Graft in Patients With Bone Loss From a Malignant Tumor Achieve Union, Function, and Complication Rate Comparably to Patients Without Bone Loss and Augmentation When Reconstructing Intercalary Resections in the Lower Extremity?. Clin. Orthop. Relat. Res..

[34] Son W.S., Lim E.J., Kim B.S., Choi W., Cho J.W., Oh J.K. (2025). Consolidation of the Anteromedial Aspect of the Tibia Is Inferior to the Other Areas in the Reconstruction of Critical-Sized Bone Defect of the Tibial Shaft Using the Induced Membrane Technique: An Analysis of 111 Serial Computed Tomography of 37 Patients. J. Orthop. Trauma.

[35] Liu K., Zhang H., Maimaiti X., Yusufu A. (2023). Bifocal versus trifocal bone transport for the management of tibial bone defects caused by fracture-related infection: A meta-analysis. J. Orthop. Surg. Res..

[36] Huang Q., Ma T., Xu Y., Lu Y., Li M., Wang Q., Ren C., Xue H., Li Z., Zhang K. (2023). Acute shortening and double-level lengthening versus bone transport for the management of large tibial bone defects after trauma and infection. Injury.

[37] Papakostidis C., Giannoudis P.V. (2023). Reconstruction of infected long bone defects: Issues and Challenges. Injury.

[38] Migliorini F., Padula G.L., Torsiello E., Spiezia F., Oliva F., Maffulli N. (2021). Strategies for large bone defect reconstruction after trauma, infections or tumour excision: A comprehensive review of the literature. Eur. J. Med. Res..

[39] Toogood P., Miclau T. (2017). Critical-Sized Bone Defects: Sequence and Planning. J. Orthop. Trauma.

[40] Corona P.S., Rosell C.C., Vicente M., Serracanta J., Tetsworth K. (2022). Three-stage limb salvage in tibial fracture related infection with composite bone and soft-tissue defect. Arch. Orthop. Trauma Surg..

[41] Zoller S.D., Cao L.A., Smith R.A., Sheppard W., Lord E.L., Hamad C.D., Ghodasra J.H., Lee C., Jeffcoat D. (2017). Staged reconstruction of diaphyseal fractures with segmental defects: Surgical and patient-reported outcomes. Injury.

[42] Masquelet A.C., Kishi T., Benko P.E. (2019). Very long-term results of post-traumatic bone defect reconstruction by the induced membrane technique. Orthop. Traumatol. Surg. Res..

[43] Molina C.S., Stinner D.J., Obremskey W.T. (2014). Treatment of Traumatic Segmental Long-Bone Defects: A Critical Analysis Review. JBJS Rev..

[44] Chen Z., Xing Y., Li X., Liu B., Liu N., Huo Y., Tian Y. (2024). 3D-printed titanium porous prosthesis combined with the Masquelet technique for the management of large femoral bone defect caused by osteomyelitis. BMC Musculoskelet. Disord..

[45] Tetsworth K., Block S., Glatt V. (2017). Putting 3D modelling and 3D printing into practice: Virtual surgery and preoperative planning to reconstruct complex post-traumatic skeletal deformities and defects. SICOT-J.

[46] Ollivier M., Bulaïd Y., Jacquet C., Pesenti S., Argenson J.n., Parratte S. (2018). Fixation augmentation using calcium-phosphate bone substitute improves outcomes of complex tibial plateau fractures. A matched, cohort study. Int. Orthop..

[47] Lefaivre K.A., Slobogean G., O’Hara N.N., O’Brien P.J. (2024). Far Cortical Locking Versus Standard Constructs for Locked Plate Fixation in the Treatment of Acute, Displaced Fractures of the Distal Femur. J. Bone Jt. Surg..

[48] Roddy E., Davis K., Wilson P., Kleweno C., Dunbar R.P., Barei D. (2025). Outcomes of Nonunion Repair for Distal Femur Fracture. J. Orthop. Trauma.

[49] Fowler T., Whitehouse M., Riddick A., Khan U., Kelly M. (2019). A Retrospective Comparative Cohort Study Comparing Temporary Internal Fixation to External Fixation at the First Stage Debridement in the Treatment of Type IIIB Open Diaphyseal Tibial Fractures. J. Orthop. Trauma.

[50] Zaheer M.U., Razzaq M.H., Aycan M.F., Mishra Y.K. (2025). AI-Assisted Design and Evaluation of SLM-Ti64 Implants for Enhanced Bone Regeneration. Adv. Healthc. Mater..

[51] Whitlock K.G., Brodke D.J., Khoury P.H., Li V., Bell A., Okhuereigbe D., Sciadini M.F., Nascone J.W., O’Toole R.V., O’Hara N.N. (2025). Ring Fixator Bone Transport Is Associated With Fewer Unplanned Major Reoperations Than Masquelet in the Treatment of Segmental Bone Defects of the Tibia. J. Orthop. Trauma.

[52] Wu W., Zhao Z., Wang Y., Yao B., Shi P., Liu M., Peng B. (2023). Clinical observation and finite element analysis of femoral stable interlocking intramedullary nail in intertrochanteric fractures. Int. Orthop..

[53] Wong K.W., Wu C.D., Chien C.S., Lee C.W., Yang T.H., Lin C.L. (2020). Patient-specific 3-dimensional printing titanium implant biomechanical evaluation for complex distal femoral open fracture reconstruction with segmental large bone defect: A nonlinear finite element analysis. Appl. Sci..

[54] Karuppudaiyan S., Singh D.K.J., Santosh V.M. (2018). Finite element analysis of scaffold for large defect in femur bone. IOP Conf. Ser. Mater. Sci. Eng..

[55] Entezari A., Zhang Z., Sue A., Sun G., Huo X., Chang C.C., Zhou S., Swain M.V., Li Q. (2019). Nondestructive characterization of bone tissue scaffolds for clinical scenarios. J. Mech. Behav. Biomed. Mater..

[56] Rana M., Chaudhuri A., Biswas J.K., Karim S.I., Datta P., Karmakar S.K., Roychowdhury A. (2021). Design of patient specific bone stiffness mimicking scaffold. Proc. Inst. Mech. Eng. Part H J. Eng. Med..

[57] Zhang T., Wei Q., Zhou H., Jing Z., Liu X., Zheng Y., Cai H., Wei F., Jiang L., Yu M. (2021). Three-dimensional-printed individualized porous implants: A new “implant-bone” interface fusion concept for large bone defect treatment. Bioact. Mater..

[58] Pobloth A.M., Checa S., Razi H., Petersen A., Weaver J.C., Schmidt-Bleek K., Windolf M., Tatai A.Á., Roth C.P., Schaser K.D. (2018). Mechanobiologically optimized 3D titanium-mesh scaffolds enhance bone regeneration in critical segmental defects in sheep. Sci. Transl. Med..

[59] Obaton A.F., Fain J., Meinel D., Tsamos A., Léonard F., Lécuelle B., Djemaï M. (2023). In vivo bone progression in and around lattice implants additively manufactured with a new titanium alloy. Appl. Sci..

[60] Karuppudaiyan S., Singh D.K.J. (2019). Design of scaffold with controlled internal architecture using fused deposition modeling (FDM). Int. J. Eng. Adv. Technol..

[61] Yavari S.A., van der Stok J., Ahmadi S., Wauthlé R., Schrooten J., Weinans H., Zadpoor A.A. (2014). Mechanical analysis of a rodent segmental bone defect model: The effects of internal fixation and implant stiffness on load transfer. J. Biomech..

[62] Lee S.S., Du X., Smit T., Bissacco E.G., Seiler D., de Wild M., Ferguson S.J. (2023). 3D-printed LEGO®-inspired titanium scaffolds for patient-specific regenerative medicine. Biomater. Adv..

[63] Vasanthanathan A., Kennedy S.M. (2023). Bio-printing of femur model: A bone substitute for biomedical research. Mater. Technol..

[64] Gabarre S., Albareda J., Gracia L., Puértolas S., Ibarz E., Herrera A. (2017). Influence of gap size, screw configuration, and nail materials in the stability of anterograde reamed intramedullary nail in femoral transverse fractures. Injury.

[65] Noyama Y., Nakano T., Ishimoto T., Sakai T., Yoshikawa H. (2013). Design and optimization of the oriented groove on the hip implant surface to promote bone microstructure integrity. Bone.

[66] Lu J., Guo S.C., Wang Q.Y., Sheng J.G., Tao S.C. (2020). J-bone graft with double locking plate: A symphony of mechanics and biology for atrophic distal femoral non-union with bone defect. J. Orthop. Surg. Res..

[67] Berven H., Brix M., Izadpanah K., Kubosch E.J., Schmal H. (2018). Comparing case-control study for treatment of proximal tibia fractures with a complete metaphyseal component in two centers with different distinct strategies: Fixation with Ilizarov frame or locking plates. J. Orthop. Surg. Res..

[68] Arabnejad S., Johnston B., Tanzer M., Pasini D. (2017). Fully porous 3D printed titanium femoral stem to reduce stress-shielding following total hip arthroplasty. J. Orthop. Res..

[69] Yamako G., Janssen D., Hanada S., Anijs T., Ochiai K., Totoribe K., Chosa E., Verdonschot N. (2017). Improving stress shielding following total hip arthroplasty by using a femoral stem made of *β* type Ti-33.6Nb-4Sn with a Young’s modulus gradation. J. Biomech..

[70] Glassman A.H., Bobyn J.D., Tanzer M. (2006). New femoral designs: Do they influence stress shielding?. Clin. Orthop. Relat. Res..

[71] Shayesteh Moghaddam N., Taheri Andani M. (2016). Metals for bone implants: Safety, design, and efficacy. Biomanufacturing Rev..

[72] Zhang Q.H., Cossey A., Tong J. (2016). Stress shielding in periprosthetic bone following a total knee replacement: Effects of implant material, design and alignment. Med. Eng. Phys..

[73] Chmielewska A., Dean D. (2024). The role of stiffness-matching in avoiding stress shielding-induced bone loss and stress concentration-induced skeletal reconstruction device failure. Acta Biomater..

[74] Ceddia M., Morizio A., Solarino G., Trentadue B. (2025). Evaluation of the Stress-Shielding Effect of a PEEK Knee Prosthesis: A Finite Element Study. Osteology.

[75] Graulich T., Kranz C., Korallus C., Örgel M., Haertlé M., Omar M., Krettek C., Panzica M. (2020). Patients outcome after distal femur- and proximal tibia replacement. Orthop. J. Sport. Med..

[76] Matar H.E., Bloch B.V., James P.J. (2021). Distal Femoral Replacements for Acute Comminuted Periprosthetic Knee Fractures: Satisfactory Clinical Outcomes at Medium-Term Follow-up. Arthroplast. Today.

[77] McAleese T., McLeod A., Keogh C., Harty J.A. (2024). Mechanical outcomes of the TFNA, InterTAN and IMHS intramedullary nailing systems for the fixation of proximal femur fractures. Injury.

[78] Stadelmann V. (2008). Prevention of Micromotion-Related Periprosthetic Bone Loss Using Local Release of Bisphosphonate: Theoretical Developments and Experimental Validations. Ph.D. Thesis.

[79] Sopher R.S., Amis A.A., Jeffers J.R.T. (2017). Total ankle replacement design and positioning affect implant-bone micromotion and bone strains. Med. Eng. Phys..

[80] Klasan A., Magill P., Frampton C., Zhu M., Young S.W. (2020). Factors predicting repeat revision and outcome after aseptic revision total knee arthroplasty: Results from the New Zealand Joint Registry. Knee Surg. Sport. Traumatol. Arthrosc..

[81] Nolte P.C., Schlentrich K., Raisch P., Jung M.K., Grützner P.A., Bischel O. (2023). Survival and Clinical Outcomes after Unconstrained Total Knee Arthroplasty for Tibial Plateau Fractures–A Retrospective Study with Minimum 4-Year Follow-Up. J. Clin. Med..

[82] Rissolio L., Sabatini L., Risitano S., Bistolfi A., Galluzzo U., Massè A., Indelli P.F. (2021). Is It the Surgeon, the Patient, or the Device? A Comprehensive Clinical and Radiological Evaluation of Factors Influencing Patient Satisfaction in 648 Total Knee Arthroplasties. J. Clin. Med..

[83] Weis S., Seifert L., Oltmanns M., Khury F., Bieger R., Faschingbauer M. (2024). The Etiology of Total Knee Arthroplasty Failure Influences on Improvement in Knee Function: A Follow-Up Study. J. Clin. Med..

[84] Wieding J., Souffrant R., Mittelmeier W., Bader R. (2013). Finite element analysis on the biomechanical stability of open porous titanium scaffolds for large segmental bone defects under physiological load conditions. Med. Eng. Phys..

[85] Bayoglu R., Okyar A.F. (2015). Implementation of boundary conditions in modeling the femur is critical for the evaluation of distal intramedullary nailing. Med. Eng. Phys..

[86] Charbonnier B., Manassero M., Bourguignon M., Decambron A., El-Hafci H., Morin C., Leon D., Bensidoum M., Corsia S., Petite H. (2020). Custom-made macroporous bioceramic implants based on triply-periodic minimal surfaces for bone defects in load-bearing sites. Acta Biomater..

[87] Kelly C.N., Lin A.S., Leguineche K.E., Shekhar S., Walsh W.R., Guldberg R.E., Gall K. (2021). Functional repair of critically sized femoral defects treated with bioinspired titanium gyroid-sheet scaffolds. J. Mech. Behav. Biomed. Mater..

[88] Lee M.C., Pan C.T., Chen W.F., Lin M.C., Shiue Y.L. (2024). Design, manufacture, and characterization of a critical-sized gradient porosity dual-material tibial defect scaffold. Bioengineering.

[89] Acar A.A., Daskalakis E., Bartolo P., Weightman A., Cooper G., Blunn G., Koc B. (2024). Customized scaffolds for large bone defects using 3D-printed modular blocks from 2D-medical images. Bio-Des. Manuf..

[90] Lu H.T., Hsu C.C., Jian Q.Q., Chen W.T. (2025). Biomechanical Study of Different Scaffold Designs for Reconstructing a Traumatic Distal Femur Defect Using Patient-Specific Computational Modeling. Comput. Model. Eng. Sci. (CMES).

[91] Benady A., Meyer S.J., Golden E., Dadia S., Levy G.K. (2023). Patient-specific Ti-6Al-4V lattice implants for critical-sized load-bearing bone defects reconstruction. Mater. Des..

[92] Bavil A., Eghan-Acquah E., Diamond L., Barrett R., Carty C., Barzan M., Nasseri A., Lloyd D., Saxby D., Feih S. (2024). Effect of different constraining boundary conditions on simulated femoral stresses and strains during gait. Sci. Rep..

[93] Scheiner S., Hellmich C. (2017). Patient-specific design of tissue engineering scaffolds, based on mathematical modeling. Advances in Ceramic Biomaterials.

[94] Laubach M., Hildebrand F., Suresh S., Wagels M., Kobbe P., Gilbert F., Kneser U., Holzapfel B.M., Hutmacher D.W. (2023). The concept of scaffold-guided bone regeneration for the treatment of long bone defects: Current clinical application and future perspective. J. Funct. Biomater..

[95] Pankaj P. (2013). Patient-specific modelling of bone and bone-implant systems: The challenges. Int. J. Numer. Methods Biomed. Eng..

[96] Hahn H., Palich W. (1970). Preliminary evaluation of porous metal surfaced titanium for orthopedic implants. J. Biomed. Mater. Res..

[97] Galante J., Rostoker W. (1973). Fiber metal composites in the fixation of skeletal prosthesis. J. Biomed. Mater. Res..

[98] Spector M. (1987). Historical review of porous-coated implants. J. Arthroplast..

[99] Fabi D., Levine B. (2012). Porous coatings on metallic implant materials. Materials for Medical Devices.

[100] Engh C.A., Bobyn J.D., Glassman A.H. (1987). Porous-coated hip replacement. The factors governing bone ingrowth, stress shielding, and clinical results. J. Bone Jt. Surg. Br..

[101] Eskola A., Vahvanen V., Santavirta S., Honkanen V., Slätis P. (1992). Porous-coated anatomic (PCA) knee arthroplasty: 3-Year results. J. Arthroplast..

[102] Nakoshi Y., Hasegawa M., Sudo A., Uchida A. (2009). A long-term follow-up study of the cementless THA with anatomic stem/HGPII cup with 22-mm head. Int. Orthop..

[103] Mow C.S., Wiedel J.D. (1998). Revision total knee arthroplasty using the porous-coated anatomic revision prosthesis: Six-to twelve-year results. J. Arthroplast..

[104] Rezapourian M., Jasiuk I., Saarna M., Hussainova I. (2023). Selective laser melted Ti6Al4V split-P TPMS lattices for bone tissue engineering. Int. J. Mech. Sci..

[105] Guo X., Ding J., Li X., Qu S., Song X., Fuh J.Y.H., Lu W.F., Zhai W. (2022). Enhancement in the mechanical behaviour of a Schwarz Primitive periodic minimal surface lattice structure design. Int. J. Mech. Sci..

[106] Li Y., Jiang D., Zhao R., Wang X., Wang L., Zhang L.C. (2024). High mechanical performance of lattice structures fabricated by additive manufacturing. Metals.

[107] Benedetti M., Du Plessis A., Ritchie R.O., Dallago M., Razavi N., Berto F. (2021). Architected cellular materials: A review on their mechanical properties towards fatigue-tolerant design and fabrication. Mater. Sci. Eng. R Rep..

[108] Rezapourian M., Kamboj N., Jasiuk I., Hussainova I. (2022). Biomimetic design of implants for long bone critical-sized defects. J. Mech. Behav. Biomed. Mater..

[109] Sahafnejad-Mohammadi I., Rahmati S., Najmoddin N., Bodaghi M. (2023). Biomimetic polycaprolactone-graphene oxide composites for 3D printing bone scaffolds. Macromol. Mater. Eng..

[110] Mall A.P., Bhandarkar V.V., Mandaloi G., Tandon P. (2024). An overview of design and development of biomimetic bone scaffolds using heterogeneous TPMS lattice structures. Arch. Comput. Methods Eng..

[111] Rezapourian M., Hussainova I. (2023). Optimal mechanical properties of Hydroxyapatite gradient Voronoi porous scaffolds for bone applications—A numerical study. J. Mech. Behav. Biomed. Mater..

[112] Qu H., Han Z., Chen Z., Tang L., Gao C., Liu K., Pan H., Fu H., Ruan C. (2021). Fractal design boosts extrusion-based 3D printing of bone-mimicking radial-gradient scaffolds. Research.

[113] Feng P., Liu L., Yang F., Min R., Wu P., Shuai C. (2024). Shape/properties collaborative intelligent manufacturing of artificial bone scaffold: Structural design and additive manufacturing process. Biofabrication.

[114] Rezapourian M., Cheloee Darabi A., Khoshbin M., Hussainova I. (2025). Multi-Objective Machine Learning Optimization of Cylindrical TPMS Lattices for Bone Implants. Biomimetics.

[115] Maskery I., Sturm L., Aremu A.O., Panesar A., Williams C.B., Tuck C.J., Wildman R.D., Ashcroft I.A., Hague R.J. (2018). Insights into the mechanical properties of several triply periodic minimal surface lattice structures made by polymer additive manufacturing. Polymer.

[116] Kadkhodapour J., Schmauder S., Sajadi F. (2022). Quality Analysis of Additively Manufactured Metals: Simulation Approaches, Processes, and Microstructure Properties.

[117] Rezapourian M., Hussainova I. (2025). Mechanical analysis of multi-surface TPMS lattices for bone applications. Proc. Est. Acad. Sci..

[118] Lu Y., Cheng L., Yang Z., Li J., Zhu H. (2020). Relationship between the morphological, mechanical and permeability properties of porous bone scaffolds and the underlying microstructure. PLoS ONE.

[119] Timercan A., Sheremetyev V., Brailovski V. (2021). Mechanical properties and fluid permeability of gyroid and diamond lattice structures for intervertebral devices: Functional requirements and comparative analysis. Sci. Technol. Adv. Mater..

[120] Toosi S., Javid-Naderi M.J., Tamayol A., Ebrahimzadeh M.H., Yaghoubian S., Mousavi Shaegh S.A. (2024). Additively manufactured porous scaffolds by design for treatment of bone defects. Front. Bioeng. Biotechnol..

[121] sadat Mirhakimi A., Dubey D., Elbestawi M.A. (2024). Laser powder bed fusion of bio-inspired metamaterials for energy absorption applications: A review. J. Mater. Res. Technol..

[122] Castrisos G., Matheus I.G., Sparks D., Lowe M., Ward N., Sehu M., Wille M.L., Phua Y., Savi F.M., Hutmacher D. (2022). Regenerative matching axial vascularisation of absorbable 3D-printed scaffold for large bone defects: A first in human series. J. Plast. Reconstr. Aesthetic Surg..

[123] Sparks D.S., Savi F.M., Dlaska C.E., Saifzadeh S., Brierly G., Ren E., Cipitria A., Reichert J.C., Wille M.L., Schuetz M.A. (2023). Convergence of scaffold-guided bone regeneration principles and microvascular tissue transfer surgery. Sci. Adv..

[124] Sparks D.S., Wiper J., Lloyd T., Wille M.L., Sehu M., Savi F.M., Ward N., Hutmacher D.W., Wagels M. (2023). Protocol for the BONE-RECON trial: A single-arm feasibility trial for critical sized lower limb BONE defect RECONstruction using the mPCL-TCP scaffold system with autologous vascularised corticoperiosteal tissue transfer. BMJ Open.

[125] Hasanabadi M., Asgari H., Azizi N., Aghajani H., Minasyan T., Toyserkani E. (2025). Towards sustainable additive manufacturing: Enhanced productivity via numerical-experimental melt pool engineering in laser powder bed fusion of Ti-alloy. J. Mater. Res. Technol..

[126] Enrique P.D., Minasyan T., Toyserkani E. (2025). Laser powder bed fusion of difficult-to-print *γ* Ni-based superalloys: A review of processing approaches, properties, and remaining challenges. Addit. Manuf..

[127] Liu L., Minasyan T., Ivanov R., Aydinyan S., Hussainova I. (2020). Selective laser melting of TiB2-Ti composite with high content of ceramic phase. Ceram. Int..

[128] Kadkhodapour J., Mirhakimi A.S., Montazerian H. (2023). Structural defects and mechanical properties of additively manufactured parts. Quality Analysis of Additively Manufactured Metals.

[129] Minasyan T., Hussainova I. (2022). Laser powder-bed fusion of ceramic particulate reinforced aluminum alloys: A review. Materials.

