# Supplementary material for: Patient-Specific Lattice Implants for Segmental Femoral and Tibial Reconstruction (Part 1): Defect Patterns, Fixation Strategies and Reconstruction Options—A Review
Source: Biomimetics (Basel). 2026 Feb 10;11(2):128. doi: 10.3390/biomimetics11020128 (PMC12938667; doi:10.3390/biomimetics11020128)
Supplement: Supplementary file 1 [file biomimetics-11-00128-s001.zip › biomimetics-4062035-supplementary.pdf]

Supplementary Material

For: Patient-specific lattice implants in femoral and tibial reconstruction (Part 1): Defect patterns, fixation strategies and reconstruction options – A review

Manuscript ID: biomimetics-4062035

Journal: Biomimetics (MDPI)

Authors: Mansoureh Rezapourian *, Anooshe Sadat Mirhakimi, Mahan Nematollahi, Tatevik Minasyan, Irina Hussainova

Contents

- Figure S1. Defect size distribution across anatomical subsegments of selected studies..

Figure S1

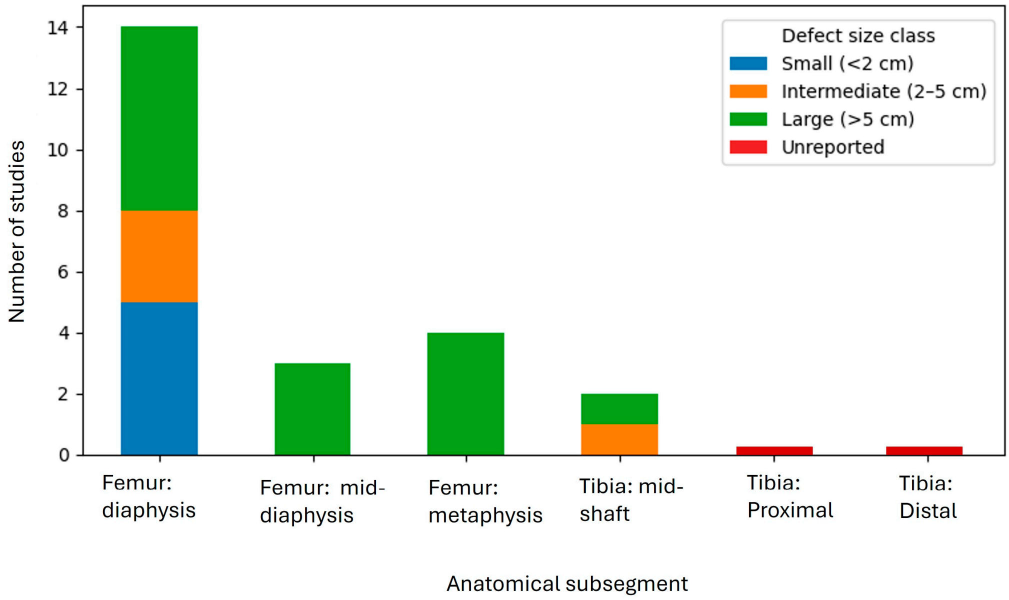

Figure S1. Distribution of defect size classes (follow figure 2; small <2 cm, intermediate 2-5 cm, large >5 cm) across anatomical subsegments (femur: diaphysis/metaphysis/meta-diaphysis; tibia: mid-shaft/proximal/distal) based on Table 2 entries [17, 18, 21-27, 53-63, 85, 87-89, 94-96].

Figure S2

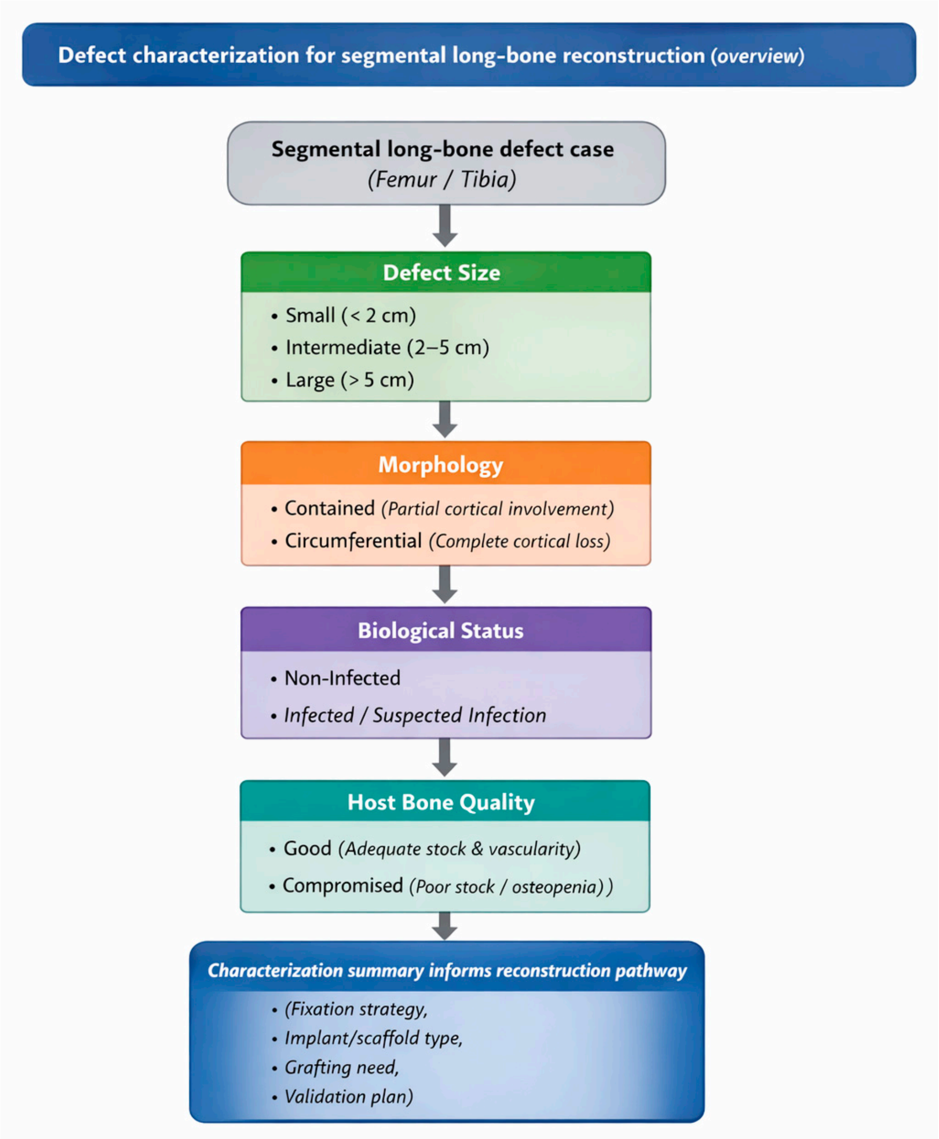

Figure S2. Flowchart for defect characterization integrating size, morphology (contained vs circumferential), biological status (infected vs non-infected), and host bone quality, consistent with Section 2.1.

Figure S3

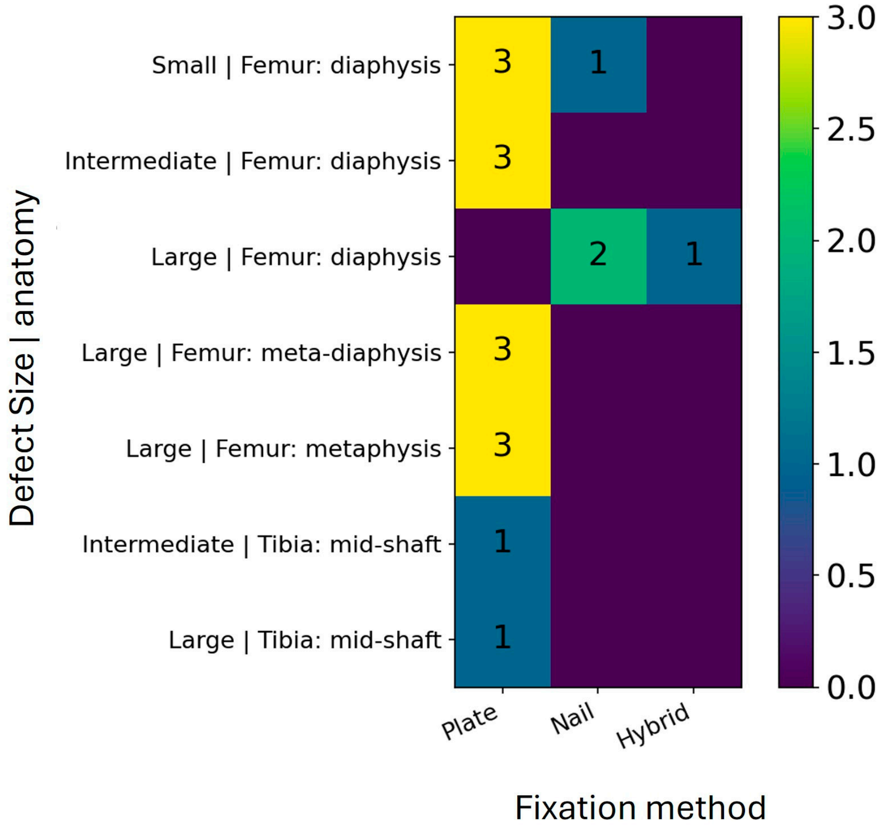

Figure S3. Heatmap showing the frequency of fixation strategies across defect size–anatomy combinations derived from Table 1. Fixation methods were grouped into plate, intramedullary nail, and hybrid (plate+nail). Defect size classes follow the thresholds used in Figure 2 (small < 2 cm, intermediate 2–5 cm, large > 5 cm), with 'large' also assigned when a study explicitly described a *large segmental defect* despite not reporting a numeric length. Entries that did not represent segmental long-bone defects (e.g., no-defect models, generic scaffold studies, intraosseous implant studies) and/or did not report a fixation construct were excluded from this frequency analysis. Only size–anatomy combinations present in Table 1 are shown (empty combinations omitted for readability).

Figure S4

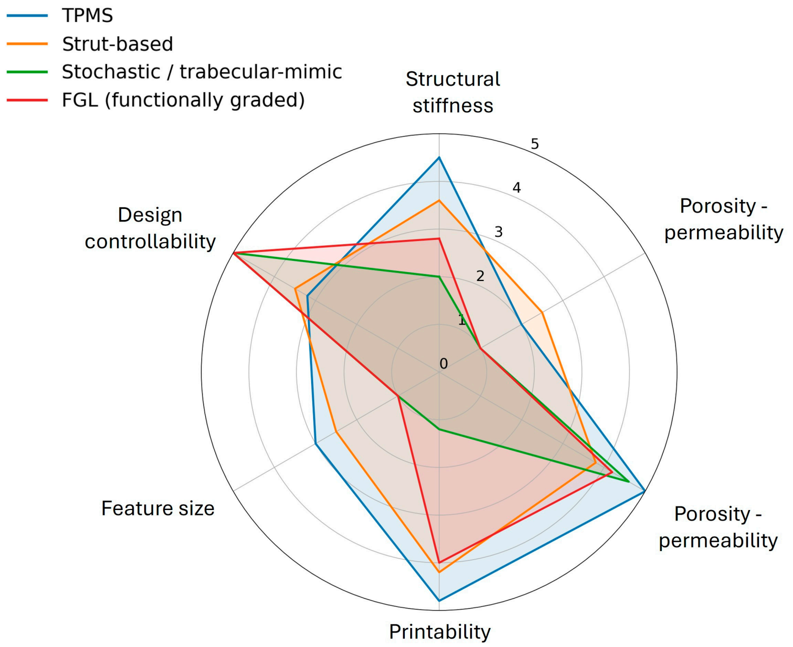

Figure S4. Semi-quantitative radar comparison of major lattice families used in segmental long-bone reconstruction studies (strut-based, TPMS, stochastic/trabecular-mimic, and functionally graded lattices). Scores (0–5) summarize how extensively the included studies *report and quantify* six criteria: stiffness-related mechanics (effective modulus and/or construct stiffness from experiments and/or FEA), fatigue/durability reporting, porosity/permeability potential, manufacturability (process feasibility and printability constraints), feature-size reporting/control, and design controllability (including gradient capability). Scores are intended as a qualitative synthesis rather than a standardized meta-analysis of absolute properties.

Figure S5

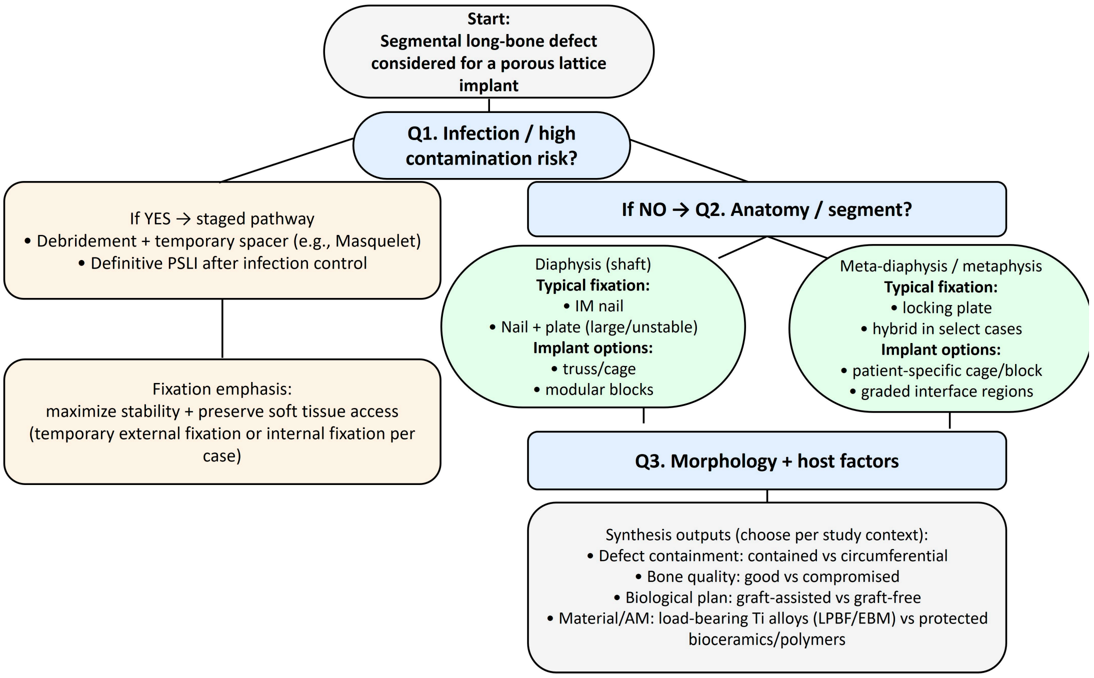

Figure S5. The schematic integrates defect size and anatomical location, defect morphology (contained vs circumferential), biological status (infected vs non-infected), and host bone quality to outline representative strategy pathways (implant architecture class, fixation concept, and material/manufacturing considerations) discussed in Section 8. The tree is intended to synthesize common decision factors reported across the reviewed literature and to support structured interpretation; it does not represent a prescriptive clinical guideline.

Table S1

Table S1. Overview of anatomical site, defect type, imaging workflow, fixation construct, and translational stage across the included segmental long-bone defect studies.

Year, Ref	Bone & segment	Defect type & size	Study model / sample	Imaging (CT/MRI; slice; segmentation)	Fixation strategy / interface intent	Clinical setting / readiness
2013, [81]	Femur distal diaphysis (segmental defect model)	Segmental defect 30 mm	Composite femur (4th-generation Sawbones); no patient/animal cohort	CT of composite femur used for Hounsfield Units (HU)-based material mapping	Angular stable lateral locking plate bridging the defect; screws modeled as beam elements anchored to plate; bone scaffold relative motion inhibited (no slip) at the interface	Bench; no in vivo/clinical
2014, [87]	Femur mid-diaphysis	6 mm segmental defect; four groups	Ex vivo cadaver femurs of male Wistar rats; animal cohort	Micro-CT used to quantify implant architecture; no in vivo imaging	Internal PEEK plate (RatFix) with 3 proximal + 3 distal screws; press-fit implant; interface objective: biomechanical load sharing (not osseointegration)	Bench only (ex vivo mechanical testing)
2015, [88]	Femur distal diaphysis	Osteotomy 5 mm in the distal one-third of the diaphysis	No patient/animal cohort	No CT/DICOM; geometry from public composite femur model (biomedtown)	Short retrograde IM nail (L=250 mm; OD 9.5 mm; ID 4.5 mm) + four interlocking pins (Ø4.5 mm); max canal gap 0.25 mm	In-silico biomechanics methodology study (no bench/clinical)
2018, [89], Case 1	Tibia mid-diaphysis	4 cm critical-size segmental defect	Large-animal in vivo: 27 operated, 24 reached endpoint (n=6 per 4 groups); 7.5 mL autologous cancellous bone graft	Monthly radiographs to 24 weeks; Second-harmonic Generation (SHG) imaging, and B-SEM at 24 weeks	4.5 mm steel Locking Compression Plate (LCP) or rigid half-shell shielding plate; interface objective: osseointegration/ingrowth via autologous bone graft (ABG) within scaffold; stiffness tuning to modulate stimulus	Preclinical in vivo; bridging prevalent with LCP groups by 24 weeks, reduced with shielding-plate groups; histology
2018, [89], Case 2	Femur/Humerus (proximal and distal), Maxillofacial (heterogeneous clinical sites)	Large segmental defects	Clinical human cohort: n=19 patients (heterogeneous ages/sex and indications: tumor	Patient-specific planning from 3D CT; radiographic follow-ups and CT at variable time points per clinical need	Plate constructs in combination with the Ti-mesh scaffold; interface objective: osseointegration/ingrowth (with graft augmentation where used)	Clinical use (case series across indications); no mechanobiologically optimized variants

Table S1. Overview of anatomical site, defect type, imaging workflow, fixation construct, and translational stage across the included segmental long-bone defect studies.

			destruction or post-traumatic nonunion)			
2018, [90]	Femur diaphysis (segmented)	Segmental defect; height = 8 mm (computational limit)	CT-based model of a healthy femur; single-subject dataset (no live cohort)	CT/DICOM, MIMICS reconstruction	Scaffold-only concept; no plate/nail; interface modeling not detailed	Bench/in-silico only
2019, [91]	Femur mid-shaft (diaphysis), Sawbones (4th gen specimens)	Cylindrical segmental defect filled with scaffold; Ø28 mm × 40 mm	Bench cohort: composite femurs (n=6)	Geometry from vendor optical scan; Digital Image Correlation (DIC) via GOM ARAMIS (two 2448×2050 CCD cameras)	Lateral 12-hole condylar plate (DePuy Synthes) + 4.5 mm self-tapping cortical screws; "rigid" vs "flexible" plate arrangements evaluated	Bench + in-silico; no animal/human implantation
2019, [22]	Femur (CAD-derived segment; no specific anatomical region)	Generic scaffold study; no clinical defect. Printed specimen size 10×10×10 mm	Bench/in-vitro only	SEM characterization (Hitachi S-570); no CT/DICOM or segmentation software reported	No fixation hardware; standalone 3D-printed PLA/β-TCP/HA scaffolds (methods only)	Bench/in-vitro readiness (accelerated degradation in PBS at 70 °C)
2019, [92]	Femur mid-diaphysis (cortical segment; model)	Segmental model (no pathology); length 30 mm	CT-derived human femur (phantom/bench; no patients); ABS scaffolds via FDM	CT (hospital source; slice not reported); segmentation in Mimics; STL exported for printing	None (standalone scaffold; no fixation)	Bench only (fabrication + porosity/mechanical evaluation)
2019, [17] , (Patient A)	Femur diaphysis (Right)	Post-traumatic infected nonunion; segmental defect length 15.2 cm (187 cc); staged interval 119 days; bone graft 123 cc	Clinical human: 53 y/o F	High-resolution CT of the temporary PMMA-spacer construct for virtual surgical planning; radiographs at weeks 0, 6, 12, 26, 52; CT at 9–12 months	Stage-1: antibiotic PMMA spacer with plate/IM nail (Masquelet); Stage-2: patient-specific 3D-printed Ti cage packed with large-volume autograft (augmented as needed) and stabilized with IM nail; interface objective: osseointegration/ingrowth	Clinical case series (Level IV); follow-up 33 months; union; no deep infection
2019, [17] (Patient B)	Femur metadiaphyseal (Right)	Open distal femoral fracture (AO/OTA 33C); segmental defect length 15.1 cm (248	Clinical human: 37 y/o M	High-resolution CT of temporary spacer construct for planning; radiographs at	Stage-1 PMMA spacer; Stage-2 patient-specific Ti cage + lateral locked plate ORIF; interface	Clinical case series (Level IV); follow-up 22 months; union; no deep infection

Table S1. Overview of anatomical site, defect type, imaging workflow, fixation construct, and translational stage across the included segmental long-bone defect studies.

		cc); staged interval 85 days; bone graft 155 cc		weeks 0, 6, 12, 26, 52; CT at 9–12 months	objective: osseointegration/ingrowth	
2019, [17] (Patient C)	Femur metadiaphyseal (Right)	Open femoral fracture; largest segmental defect length 18.4 cm (292 cc); staged interval 108 days; bone graft 239 cc	Clinical human: 56 y/o M	High-resolution CT of temporary spacer construct for planning; radiographs at weeks 0, 6, 12, 26, 52; CT at 9–12 months	Stage-1 PMMA spacer; Stage-2 patient-specific Ti cage + lateral locked plate (ORIF); interface objective: osseointegration/ingrowth	Clinical case series (Level IV); follow-up 25 months; union
2019, [17] (Patient D)	Femur metadiaphyseal (Left)	Post-traumatic infected nonunion; segmental defect length 10.3 cm (114 cc); staged interval 106 days; bone graft 77 cc	Clinical human: 73 y/o F	High-resolution CT of temporary spacer construct for planning; radiographs at weeks 0, 6, 12, 26, 52; CT at 9–12 months	Stage-1 PMMA spacer; Stage-2 patient-specific Ti cage + lateral locked plate (ORIF); interface objective: osseointegration/ingrowth	Clinical case series (Level IV); follow-up 17 months; union; no deep infection
2019, [17] (Patient E)	Femur diaphysis (Right)	Open femoral fracture; segmental defect length 11.1 cm (121 cc); staged interval 83 days; bone graft 72 cc	Clinical human: 26 y/o M	High-resolution CT of temporary spacer construct for planning; radiographs at weeks 0, 6, 12, 26, 52; CT at 9–12 months	Stage-1 PMMA spacer; Stage-2 patient-specific Ti cage + IM nail; interface objective: osseointegration/ingrowth	Clinical case series (Level IV); follow-up 12 months; union; no deep infection
2020, [93]	Femur mid-diaphysis (rat)	Segmental diaphyseal defect, 3 mm	Animal cohort (rat): Lewis rats, 12–15 weeks (N=8 per architecture)	X-ray + micro-CT for implant design and longitudinal assessment; CT/DICOM slice specs and segmentation software not reported	8-hole PEEK locking plate (RatFix) with six screws; no screws through implant	In vivo animal model with micro-CT, histology, SEM-EDX; not human clinical
2020, [94]	Femur distal (right), lateral condyle region	Complex open fracture with large segmental bone defect; open wound >15 cm; intraoperative bone loss >10 cm	Human single case: 17 y/o M	CT-based bilateral femur reconstruction; cortical/cancellous contours extracted; intact left mirrored to right	PSI titanium implant with proximal cylinder tube, 1.5-mm proximal/distal shells for ingrowth, outer lattice mesh (10×10 mm, 1.5 mm thick) for bone graft; protruding stem; transverse (proximal) and oblique (distal) implant screws; lateral pre-contoured locking plate with 0.5-mm gap	In-silico surgical planning; reconstruction planned after soft-tissue recovery; no in vivo implantation reported

Table S1. Overview of anatomical site, defect type, imaging workflow, fixation construct, and translational stage across the included segmental long-bone defect studies.

2021, [83]	Femur (greater trochanter, diaphysis, epicondyle; whole-model sections)	Stiffness-matching implant concept; no explicit segmental defect size (resection-replacement scenario)	Single subject: 55 y/o F	CT/DICOM 512×512; pixel 0.6445 mm; slice thickness 1 mm; segmentation in MIMICS v17; FE in ANSYS 19	No plate/nail; implants bonded to bone in FE (bonded contact); AM prototypes; objective: stiffness matching for osseointegration-friendly mechanics	Bench/in-silico with compression tests (no in vivo/clinical)
2021, [80]	Femur diaphyseal critical segmental defect (rat)	Segmental defect (CSBD), L=8 mm	Animal cohort: rat femora	X-ray + micro-CT over 12 weeks; micro-CT used for scaffold morphology/porosity (no patient CT/segmentation)	Plate fixation across defect; scaffold used as carrier for BMP-2 in alginate hydrogel (0.5 mg/cm³) to encourage ingrowth	Preclinical in vivo (rat); functional repair shown with imaging and histology endpoints
2021, [21]	Femur (generic model from CT)	Generic scaffold investigation; no explicit defect size	In-silico/bench only; CT of a 37 y/o patient used for modeling (no human/animal cohort)	CT to 3D in 3D Slicer 4.11; mesh prep in Meshmixer; HyperMesh 2020; FEA in Abaqus/CAE 2020	No fixation hardware; standalone SLA-printed scaffolds; UV-cured biodegradable resin with/without 5% hydroxyapatite (HA) + 5% calcium pyrophosphate (CPP); ASTM D638/D695 used	Bench/FEA only; compression and tensile tests reported
2021, [78]	Femur diaphysis (left)	11 cm segmental defect (post-traumatic osteomyelitis; non-union)	Human patient: 64 y/o F	Radiographic follow-up immediately and at 2, 5, 8, 14, 20 months; infection control with staged debridements; CT for implant design (slice not reported)	Patient-specific implant + intramedullary nail; objective: ingrowth without autograft and allograft	Clinical human case within same series; 20 month follow-up
2021, [78], Case 1	Sheep femur mid-diaphysis (right)	4 cm critical-size segmental defect	Large-animal in vivo (sheep)	Radiographs at 1, 3, 6 months; high-resolution micro-CT (20 µm, Inveon MM); CT-based FEA: Mimics Research 20.0 → Abaqus 6.14; three-point bending after screw removal	Lateral plate + screws integrated with porous implant; objective: osseointegration/ingrowth; three-point bending bench test	Preclinical in vivo animal study + bench/FEA tests

Table S1. Overview of anatomical site, defect type, imaging workflow, fixation construct, and translational stage across the included segmental long-bone defect studies.

2021, [78], Case 2	Sheep femur mid-diaphysis (right)	4 cm critical-size segmental defect; endpoints at 1, 3, 6 months	Large-animal in vivo: Small Tail Han sheep (17±2.9 mo; 47.8±5.3 kg), randomized across time-point groups	High-res micro-CT 20 µm (Inveon MM); 3D reconstruction in Inveon Research Workplace; screw removal before micro-CT/bench tests	Screw–plate system unified with porous implant for immediate stability; no grafts/osteoinductive agents; objective: ingrowth	Preclinical in vivo + bench/FEA; progressive intra-porous/peri-implant bone formation quantified by Bone Volume Fraction (BVF)
2022, [23]	Femur (segmental site; model-defined)	Segmental critical-size defect, 50 mm	Single adult subject CT used to build femur model; no live cohort (in-silico)	CT/DICOM; segmentation	Scaffold-only (no plate/nail); interface objective: osseointegration/stress-sharing; stress-shielding reduction shown vs solid	In-silico mechanical evaluation (FE compression + physiological hip/muscle loads); no animal/human implantation
2022, [26]	Distal lateral femur (above epiphyseal plate); osteosarcoma-related large defect (LW)	LW defect defined anatomically (depth = 3/4·X; located at Y/3 of femur length); no absolute mm value reported	Composite femur (Sawbones, 4th Gen., 17 PCF); n=3 implant vs n=3 cement; patient CT used to size implant; no human/animal subjects	Patient CT → CAD model; FE in ANSYS v19.0; CT slice/segmentation software not reported	PSI Ti-6Al-4V scaffold with outer surface lattice (5×5 mm grid; 10×10 mm for larger bones) + lateral locking plate (236 mm) and screws (Ø5×26 mm proximal; Ø5×60 mm distal); countersunk screw holes; objective: ingrowth/allograft fill	Bench + FE only: nine-stage cyclic loading (0.5–5× body weight), 20,000 cycles/stage (total 180,000); no in vivo/clinical
2023, [24]	Femur segmental (intercalary) scaffold for large femoral defects	Large segmental defect; no single numeric length reported	Single-subject CT dataset (40 y/o M, 75 kg); in-silico only—no animal/human cohort	CT/DICOM; segmentation in Mimics 21.0; surface repair in Magics 22.0	Personalized integrated fixation device (lateral approach) with two rows of threaded holes at 60 deg on lateral and anterior faces; upper/lower arms 50 mm; 4.5 mm tapping screws; compared against plate fixation; objective: osseointegration/ingrowth via graded porosity	Bench/in-silico FE only; no printed implant or animal studies reported (stated limitations)

Table S1. Overview of anatomical site, defect type, imaging workflow, fixation construct, and translational stage across the included segmental long-bone defect studies.

2023, [95]	Concept only (schematic apps shown for mandible, spine, femur; no specific bone segment studied)	Modular ATS blocks (8×8×5.8 mm per unit; SP/UP porous variants) assembled to fit arbitrary defect sizes	Bench only: in-vitro cells (MC3T3-E1 pre-osteoblasts; hMSCs); no animal/human cohort	Scaffold micro-CT; CLSM topography (283×283 µm); no patient CT/DICOM	No fixation (concept study); interface objective: promote osseointegration via porosity + double acid-etched nano-roughness	Bench only (in-vitro + mechanical + FEA); readiness: preclinical concept
2023, [96]	Femur (whole bone; full-length anatomical model)	No defect; anatomical femur model	Phantom/model only; geometry from CT of a 33 y/o M	CT-DICOM; segmentation	No fixation (bench model); interface objective: N/A	Bench only (in-vitro phantom + FEA + compression test); concept/model study
2023, [18]	Sheep metatarsus mid-diaphysis	15 mm segmental defect; patient-specific outer scaffold with distal marrow coupler; cancellous autograft packed in grafting cavity	Large-animal in vivo: n=8 adult sheep (S1–S8)	Pre-op CT DICOM; segmentation; radiographs; per-animal CT checkpoints reported	Ilizarov-type external fixator (two circular frames; six 4 mm Schanz pins per frame; instrumented connecting bars; stiffness Kf = 593 N/mm) + robocast HA scaffold; interface objective: osseointegration/ingrowth	Preclinical in vivo large-animal study with longitudinal mechanical + imaging monitoring
2023, [97]	Tibia and metatarsal (intraosseous sites)	No segmental defect; intraosseous cylindrical lattice implants (Ø6 mm; total height 8–12.4 mm)	In vivo large animal: sheep	Post-explant 3D XCT; ML segmentation (Ilastik + two UNets); BIC measured on 1-mm sagittal sections (pixel size ≈ 6.8 µm)	No plate/nail; implants placed intraosseously without additional fixation; interface objective: osseointegration (Bone Implant Interface (BII) < 10 µm; Bone Implant Contact (BIC) up to ~95%)	Preclinical in vivo animal study; 12-week implantation with imaging/quantification
2024, [79]	Tibia mid-shaft (diaphysis), rabbit model	Critical-sized tibial cortical defect	Geometry from New Zealand white rabbit tibia; no live-animal implantation (bench design/FE + prototype)	CT used to reconstruct tibia; CAD via Geomagic Studio for SolidWorks 2017; slice thickness not stated	Curved Ti bone plate + screws integrated with porous Ti-6Al-4V ELI cage (radial gradient porosity); interface objective: load-sharing with ingrowth (HA fill considered in design)	Bench/in-silico and prototype manufacturing (LPBF/SLM in original paper); no in vivo

Table S1. Overview of anatomical site, defect type, imaging workflow, fixation construct, and translational stage across the included segmental long-bone defect studies.

2024, [98]	Femur shaft (diaphysis); defect zone assumed from 20 mm below the minor trochanter to 20 mm above the start of the condyle	Large segmental shaft defects; scenarios shown at 67.60 mm (distal shaft) and 109.20 mm (mid-shaft)	No human/animal cohort	2D medical images (X-rays) used; algorithm reconstructs 3D from 2D; no CT slice or segmentation specs	Modular scaffold blocks assembled with an intramedullary femoral nail for stability and guidance	Bench/prototyping only; no in vivo or clinical series
2024, [28]	Femur distal (condylar region; model + animal)	Model: distal femur defect, 25 mm height created 55 mm from joint surface; Animal: intraosseous cylindrical implant Ø4.2×10 mm in distal lateral femur	Bench + in-vitro cells; in vivo large-animal: Lanyu pigs n=6 (n=3 OLS lattice; n=3 solid)	CT for creating geometry; post-op CT and micro-CT for osseointegration at 2, 4, 8, 12 weeks; micro-CT 9 µm; CAD in Creo; FEA with convergence check	Lateral reconstruction plate + screws; one screw links implant to plate; interface objective: osseointegration via surface lattice (target bone strain 2000–2250 µε)	Preclinical (FE + bench + in-vitro + in vivo animal)
2025, [76]	Femur distal defect (patient-specific femur model)	Traumatic or tumor-like distal femur bone loss; defect length 82 mm	Patient-specific from NIH Visible Human male right femur CT; in silico only (no live cohort)	CT; segmentation (Amira Avizo); STL processing (Geomagic Freeform/Wrap); HU-E material mapping; FE in ANSYS Workbench	Scaffold and two connection plates and two locking screws (buttress thread; OD 4 mm, ID 3 mm, pitch 1.48 mm; lengths 40 mm proximal and 55 mm distal); Ti-6Al-4V; interfaces assumed bonded; single leg stance loads; distal external surfaces fixed	In silico finite element analysis only
